# Blood biomarkers of vascular dysfunction in small vessel disease progression: Insights from a longitudinal neuroimaging study

**DOI:** 10.1002/alz.70152

**Published:** 2025-04-25

**Authors:** Daniela Jaime Garcia, Una Clancy, Carmen Arteaga, Maria C. Valdés‐Hernandez, Francesca M. Chappell, Angela C. C. Jochems, Yajun Cheng, Junfang Zhang, Michael J. Thrippleton, Michael S. Stringer, Emilie Sleight, Ellen V. Backhouse, Stewart Wiseman, Rosalind Brown, Fergus N. Doubal, Axel Montagne, Joanna M. Wardlaw, The MSS3 Study Group

**Affiliations:** ^1^ Centre for Clinical Brain Sciences School of Clinical Sciences College of Medicine and Veterinary Medicine The University of Edinburgh Edinburgh UK; ^2^ UK Dementia Research Institute at the University of Edinburgh Edinburgh UK; ^3^ Department of Neurology West China Hospital Sichuan University Chengdu China; ^4^ Department of Neurology & Institute of Neurology Ruijin Hospital affiliated with Shanghai Jiao Tong University School of Medicine Shanghai China; ^5^ Edinburgh Imaging Facility (Royal Infirmary of Edinburgh) University of Edinburgh Edinburgh UK

**Keywords:** biomarkers, blood‐brain barrier, cerebrovascular dysfunction, cerebrovascular reactivity, cognitive impairment, endothelial dysfunction, pericyte, small vessel disease, vascular dementia

## Abstract

**INTRODUCTION:**

This study explored the relationship between blood biomarkers of cerebrovascular function and small vessel disease (SVD) neuroimaging markers and cognitive outcomes in highly‐phenotyped participants.

**METHODS:**

We conducted cross‐sectional and 1‐year longitudinal analyses on 181 patients with mild ischemic stroke, enriched for SVD features. We examined relationships between a panel of 13 blood biomarkers and magnetic resonance imaging (MRI) markers of SVD (structural lesions, diffusion‐weighted imaging [DWI]‐positive lesions, blood‐brain barrier (BBB) permeability, and cerebrovascular reactivity (CVR), and cognition.

**RESULTS:**

In linear mixed models, vascular endothelial growth factor was significantly associated with incident DWI‐positive lesions over 1 year. Intercellular adhesion molecule‐1 was linked with lower CVR while platelet‐derived growth factor‐subunit B and Endothelin‐1 were associated with higher CVR. Platelet‐Selectin levels were associated with mild cognitive impairment at 1 year.

**DISCUSSION:**

Our results support the role of endothelial and pericyte dysfunction in SVD burden and progression and suggest that specific biomarkers relate to distinct SVD manifestations.

**Highlights:**

- Small vessel disease (SVD) lacks specific or predictive biomarker signatures.
- Vascular endothelial growth factor levels were linked to incident lesions detected over 1 year.
- Circulating intercellular adhesion molecule‐1 related to lower cerebrovascular reactivity.
- Platelet‐selectin levels were associated with mild cognitive impairment longitudinally.
- These findings could help stratify patients at high‐risk of rapid‐progression SVD.

## 1 BACKGROUND

Cerebral small vessel disease (SVD) is a highly prevalent disorder of the small brain blood vessels and a common cause of stroke and dementia, contributing to approximately 45% of all dementia cases worldwide.^1^ SVD is characterized by structural and functional alterations in cerebral arterioles, venules, capillaries, and surrounding tissue. While clinical manifestations of SVD are highly varied, they can include lacunar or hemorrhagic stroke, cognitive decline, neuropsychiatric symptoms, and mobility problems.^2^, ^3^, ^4^ SVD is distinguished by various signature lesions visible on neuroimaging, including white matter hyperintensities (WMH), lacunes, microbleeds, and enlarged perivascular spaces (PVS). While many aspects of the underlying pathogenesis of SVD remain uncertain, numerous lines of evidence implicate endothelial and pericyte dysfunction, inflammation, and a compromised blood‐brain barrier (BBB), which may precede visible brain lesions.^5^

Despite compelling evidence advocating for the role of endothelial and pericyte dysfunction in the pathogenesis of SVD, there are currently no blood biomarkers reliably capable of characterizing the disease and results from previous studies remain inconsistent.^6^ Some of these discrepancies may arise due to the inherent complexity underlying SVD mechanisms as well as variations introduced by study design and sample heterogeneity. Some results may be confounded by an acute response to ischemic injury by comparing vascular function markers in lacunar stroke patients within the hyper‐acute stroke stage to healthy controls or effects of prescribed drugs. Others exclude patients with a history of stroke or mild cognitive impairment (MCI) altogether, limiting the scope of SVD severity representation or relevance. Importantly, few studies include a wide range of SVD‐relevant features, including cognitive decline, BBB permeability, and cerebrovascular reactivity (CVR), as well as diverse lesion types, because although WMH are more commonly observed, it remains possible that distinct lesions represent different stages of a dynamic disease process.^3^

**FIGURE 1 alz70152-fig-0001:**
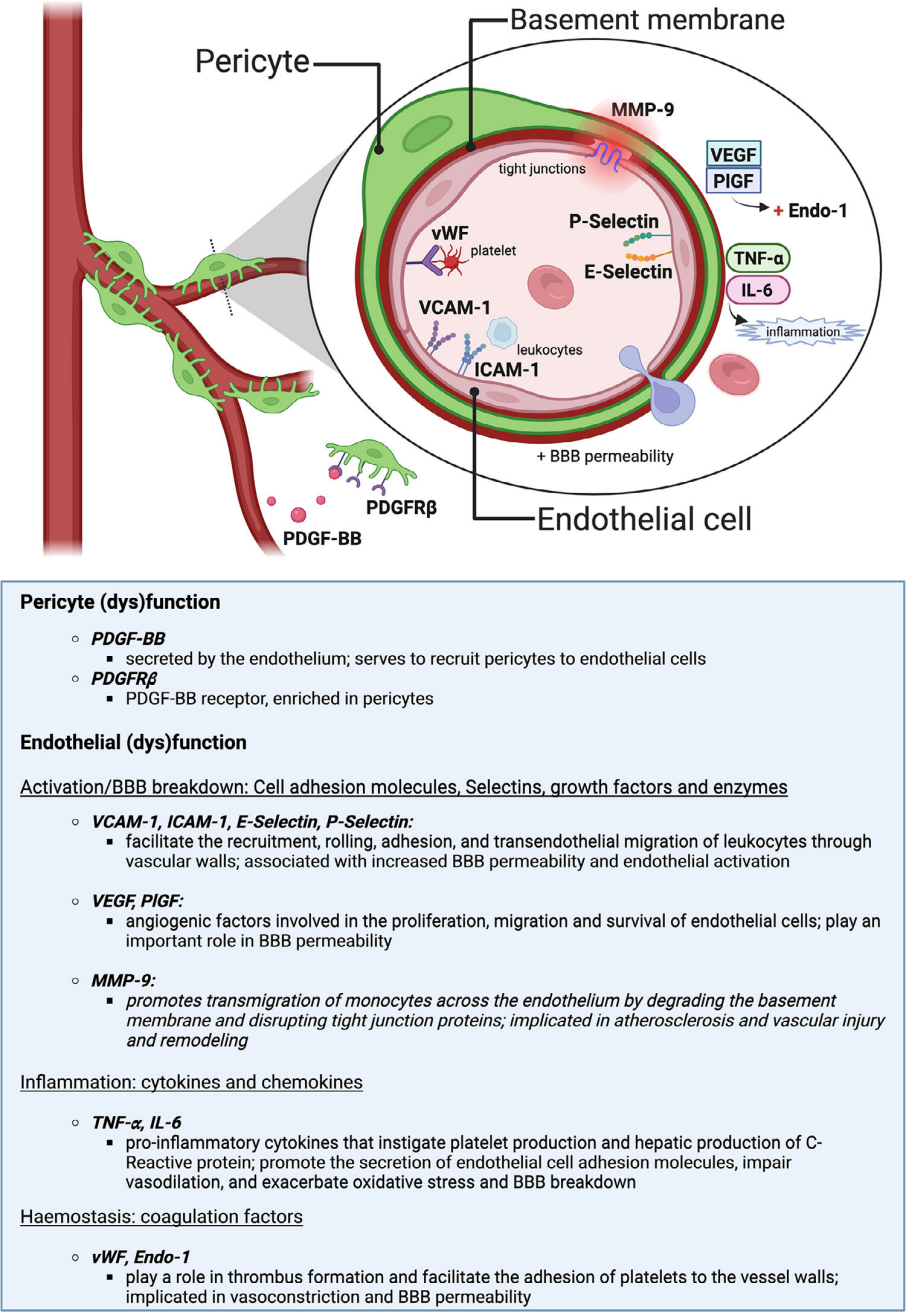
Panel of serum biomarkers extracted and their putative involvement in pericyte and endothelial cell (dys)function in the cerebral small vessels. BBB, blood‐brain barrier; E‐Selectin, endothelial‐selectin; ICAM‐1, intercellular adhesion molecule 1; IL‐6, Interleukin 6; MMP‐9, matrix metalloproteinase 9; P‐Selectin, platelet‐selectin; PDGF‐BB, platelet derived growth factor subunit B; PDGFRβ, platelet derived growth factor receptor beta; PlGF, placental growth factor; TNF‐α, tumor necrosis factor alpha; VCAM‐1, vascular cell adhesion molecule 1; VEGF, vascular endothelial growth factor; vWF, von Willebrand Factor; Endo‐1, Endothelin‐1.

We aimed to examine the relationship between various biomarkers of vascular, endothelial, and pericyte function and (1) structural and vascular functional MRI markers of SVD, including WMH, lacunes, microbleeds, summary SVD score, BBB permeability, and CVR, (2) cognitive performance (including the executive function domain) and MCI in cross‐sectional and longitudinal exploratory analyses with patients with minor, non‐disabling ischemic stroke, enriched for SVD features. A concise list of the biomarkers extracted, their roles, and their putative involvement in cerebrovascular dysfunction is presented in Figure 1 and includes: platelet derived growth factor receptor beta (PDGFRβ), platelet derived growth factor subunit B (PDGF‐BB), vascular cell adhesion molecule‐1 (VCAM‐1), intercellular adhesion molecule‐1 (ICAM‐1), platelet selectin (P‐Selectin), endothelial selectin (E‐Selectin), endothelin‐1 (Endo‐1), tumor necrosis factor alpha (TNF‐α), von Willebrand factor (vWF), matrix metalloproteinase‐9 (MMP‐9), interleukin‐6 (IL‐6), vascular endothelial growth factor (VEGF), and placental growth factor (PlGF).

## 2 METHODS

### 2.1 Participants and design

We prospectively recruited 229 patients presenting to the Lothian stroke services with acute lacunar or minor cortical ischemic stroke from August 2018 to December 2021 into an observational longitudinal cohort study.^7^ All participants provided written informed consent according to the principles expressed in the Declaration of Helsinki. This study was granted ethical approval by Lothian Ethics Medical Research Committee (REC 09/81,101/54) and NHS Lothian R&D Office (2009/W/NEU/14).

Participants were recruited between 1 and 3 months post‐stroke. All clinical evaluations, including stroke diagnosis and subtyping, were conducted by specialized stroke physicians and neuroradiologists. Patients with MRI contraindications or severe neurological, cardiac, or respiratory conditions were excluded from the study. Minor stroke was characterized by a modified Rankin Score (mRS) of ≤2 at recruitment.^8^, ^9^ All patients received guideline stroke prevention treatments as clinically appropriate including antihypertensive, lipid lowering, and antiplatelet drugs.

Patients attended a baseline visit that included serology, brain MRI, cognitive, and clinical assessments. Structural MRI and cognitive assessments were repeated 1 year later.^7^ In addition, those with lacunar stroke or moderate‐to‐severe WMH were invited for additional follow‐up scans at 2–3 and 4–6 months. Details of relevant clinical events occurring throughout the study period, including recurrent stroke, transient ischemic attack, or suspected stroke, were sought.

### 2.2 MRI acquisition

Detailed neuroimaging protocols are described elsewhere.^7^ Briefly, participants underwent 3 T brain MRI (MAGNETOM Prisma, Siemens Healthcare) at baseline and 1 year. Images were acquired using a 32‐channel head coil (Siemens Healthcare, Erlangen, Germany). The following sequences were acquired: 3D T1‐weighted (1 mm^3^ isotropic), T2‐weighted (0.9 mm^3^ isotropic), FLAIR (1 mm^3^ isotropic), multi‐shell diffusion weighted imaging (b = 2000 (64 orientations), 1000 (64), 600 (6), 200 (3), and 0 (15) s/mm^2^; 2 mm^3^ isotropic voxels), susceptibility‐weighted imaging (SWI/SWAN/GRE; 0.6/0.6/3.0 mm^3^), and, at baseline, a 21‐min dynamic contrast‐enhanced (DCE)‐MRI protocol (2.0 mm^3^) including a gadolinium‐based contrast agent (gadobutrol) injection to assess BBB integrity, except in patients with estimated glomerular filtration rate (eGFR) levels of <30 mL/min.^10^, ^11^ DCE sequence was followed by DESPOT1‐HIFI sequence to determine voxel‐wise pre‐contrast T1 values.^12^ At baseline, we also acquired 2D gradient‐echo EPI images (2.5 mm^3^ isotropic) in conjunction with a hypercapnic challenge to assess CVR, consisting of a 12‐min protocol alternating between medical air and hypercapnic gas (6% CO_2_, 21% O_2_, 73% N_2_).^13^ Heart rate, respiration rate, peripheral oxygen saturation levels, and end‐tidal CO_2_ (EtCO_2_) were closely monitored during this procedure.

**RESEARCH IN CONTEXT**

1. **Systematic review**: The authors reviewed the literature using traditional (e.g., PubMed) sources. Recently published evidence strongly supports the role of pericyte and endothelial cell dysfunction in the pathogenesis of small vessel disease (SVD). However, results from studies examining pericyte and vascular biomarkers and SVD are inconsistent, and no sole biomarker or biomarker signature can consistently characterize disease or predict its progression.
2. **Interpretation**: A wide range of vascular and pericyte function blood biomarkers were measured in patients with mild ischemic stroke, enriched for SVD features. We provide a holistic analysis of how these biomarkers relate to distinct manifestations of disease longitudinally, including structural and vascular functional MRI markers and cognition.
3. **Future directions**: Establishing disease‐specific biomarkers could facilitate efforts to uncover pathophysiology and develop therapeutic interventions and evaluate their effectiveness. Results from these exploratory analyses should be validated in larger, more diverse populations, ideally with an extensive follow‐up period.

### 2.3 MRI processing and analysis

MRI data were processed computationally and semi‐automatically using validated methods.^7^, ^14^ Intracranial volumes (ICVs) were computationally generated, checked for accuracy and edited manually[Fig alz70152-fig-0001] when required. WMH volumes were extracted from fluid‐attenuated inversion recovery (FLAIR) images.^14^ Index acute, old, and incident infarcts were manually segmented and excluded from total WMH volumes by an experienced image analyst.

Scans were assessed for acute ischemic lesions, including incident small subcortical DWI‐positive lesions, old infarcts, lacunes, microbleeds, summary SVD scores (assigning one point for: (1) Fazekas score of three in the periventricular white matter or ≥2 in deep white matter; (2) lacunes present; (3) microbleeds present; (4) over 10 counts of enlarged perivascular spaces in the basal ganglia), and Fazekas scores (periventricular white matter and deep white matter) by trained raters and checked by an experienced neuroradiologist according to STRIVE criteria using validated scales.^14^, ^15^, ^16^, ^17^, ^18^, ^19^ To assess BBB permeability, we derived values for permeability‐surface area product (PS) and blood plasma volume fraction (V_p_) for each voxel and region using validated in‐house processing scripts and DCE‐MRI sequences. Full details on DCE‐MRI acquisition protocol are described elsewhere.^7^, ^20^ CVR was quantified as the regression coefficient associated with a time‐shifted EtCO_2_ profile divided by the baseline BOLD signal (mean BOLD intensity across the first volumes of the first medical air block) multiplied by 100. CVR corresponded to the relative change in BOLD signal per unit change in EtCO_2_ and is reported as %/mmHg in normal‐appearing white matter (NAWM), deep gray matter, and within WMH. Additional details are published.^13^

### 2.4 Blood biomarkers

We collected a 19 mL venous blood sample from participants at the baseline visit. Within 1 hour of collection, samples were centrifuged (at 1000 × *g* for 10 min for serum samples and 2000 × *g* for 15 min for plasma samples) to isolate serum and plasma before being stored at –80°C. Using commercially available enzyme‐linked immunosorbent assay (ELISA) kits, we extracted the following biomarkers from patient serum samples: PDGFRβ (Abcam‐ab252357), PDGF‐BB (Abcam‐ab100624), VCAM‐1 (Abcam‐ab223591), ICAM‐1 (R&D‐DY720), P‐Selectin (R&D‐DPSE00), E‐Selectin (R&D‐DSLE00), Endo‐1 (R&D‐DET100), TNF‐α (R&D‐HSTA00E), vWF (Abcam‐ab108918), MMP‐9 (R&D‐DMP900), IL‐6 (R&D‐HS600C), VEGF (R&D‐DVE00), and PlGF (R&D‐HSPG00). Plasma samples were used in lieu of serum samples for seven participants (>4%) as the latter were unavailable. According to ELISA kit instructions, either serum or plasma can be used, with the exception for the E‐Selectin and MMP‐9 kits. For those two, we did not include the seven plasma samples. Dilution testing was conducted in both serum and plasma separately. Additionally, we standardized the biomarkers to a mean of 0 and a SD of 1, thus further minimizing any extraneous variation due to processing (see Section 2.6). Additional details pertaining to the assay protocol, including dilutions, and calculated inter‐ and intra‐assay %, can be found in supplemental materials, Appendix A.

### 2.5 Clinical and cognitive assessments

Study procedures are described in detail elsewhere.^7^ Participants provided medical history confirmed by a physician and supplemented by hospital medical records, including diabetes mellitus, hypertension, and hypercholesterolemia, and provided details on smoking status and history. Stroke subtype (lacunar or cortical) was determined by reaching consensus between an expert panel of stroke physicians and neuroradiologists based on presenting symptoms and diagnostic neuroimaging or relevant investigations.^7^, ^21^ Cortical strokes served as a control group for lacunar stroke and are more relevant than non‐stroke controls, as individuals with cortical strokes receive similar secondary prevention medications that can influence endothelial function and blood markers. Cognition was assessed using the Montreal Cognitive Assessment (MoCA) at baseline and again at 1 year. A cut‐off score of 26 out of 30 (including the addition of a one‐point educational adjustment for individuals with ≤12 years of education) was used to establish MCI classification.^22^ Participants also completed the Trail Making Test (TMT) parts A and B  at baseline and 1 year. We derived the B/A ratio from this task to assess executive functioning^23^; B/A ratios of >3 indicate executive dysfunction. We also quantified premorbid/peak adult intelligence by measuring number of errors in the National Adult Reading Test (NART).^24^

### 2.6 Statistical analyses

All analyses were performed using R (version 4.4.1).^25^ We expressed WMH volumes as % ICV to adjust for variations in individual head size. WMH volumes as % ICV were log‐transformed to improve model fit and ensure normality of residuals. To reduce the number of predictors and avoid model overfitting, we assigned equal weight to hypertension, hypercholesterolemia, diabetes, and smoking history (current smoker or ex‐smoker within 1 year) in the calculation of a baseline vascular risk factor composite score. Models were assessed for assumptions and fit by checking the normality of residuals, heteroscedasticity, and collinearity. Blood biomarker levels were standardized (mean = 0, SD = 1) to improve model convergence and facilitate inter‐model coefficient comparisons. The nature of this study was exploratory and hypothesis‐generating, thus *p*‐values were not adjusted for multiple comparisons. Findings should be interpreted as preliminary.

### 2.7 Cross‐sectional analyses

All models were adjusted for age, sex, and vascular risk factor composite score. Models with neuroimaging‐related outcome variables also included stroke subtype and the National Institutes of Health Stroke Scale (NIHSS) at baseline as covariates, while models with cognition‐related outcome variables were additionally adjusted for premorbid intelligence and baseline WMH volume as % ICV. Adjustment for stroke subtype (lacunar vs. cortical) accounts for stroke mechanism and prescribed medication.

Linear regression was used to assess the relationship between individual standardized biomarkers and log transformed WMH volume, continuous MoCA score, PS, V_p_, and CVR (in NAWM, deep gray matter, and within WMH, separately) as outcome variables. Logistic regression was used to examine the relationship between the standardized biomarkers and the prevalence of lacunes and microbleeds at baseline (≥1), the incidence of DWI‐hyperintense lesions (≥1) during the 1‐year follow‐up period, and MCI prevalence. Finally, to assess the relationship between individual standardized biomarkers and Fazekas score and summary SVD score, we used ordinal regression models adjusted for the variables outlined above.

### 2.8 Longitudinal analyses

Longitudinal analyses were performed using linear mixed‐effects models (lme4, R, R Foundation for Statistical Computing, Vienna, Austria) with individual participants fitted as random effects to account for intraindividual trajectories.^26^ Models with longitudinal WMH volumes as the outcome variable were adjusted for age, sex, vascular risk factor composite score, stroke subtype, and baseline NIHSS; models with longitudinal MoCA scores and TMT ratio as outcome variables included age, sex, vascular risk factor composite score, premorbid intelligence, and baseline WMH volume as % ICV as covariates. Longitudinal analyses using SVD summary score, Fazekas score, and lacune and microbleed prevalence were not conducted here, as these measurements might not effectively capture subtle changes occurring over a 1‐year period.

## 3 RESULTS

**FIGURE 2:**
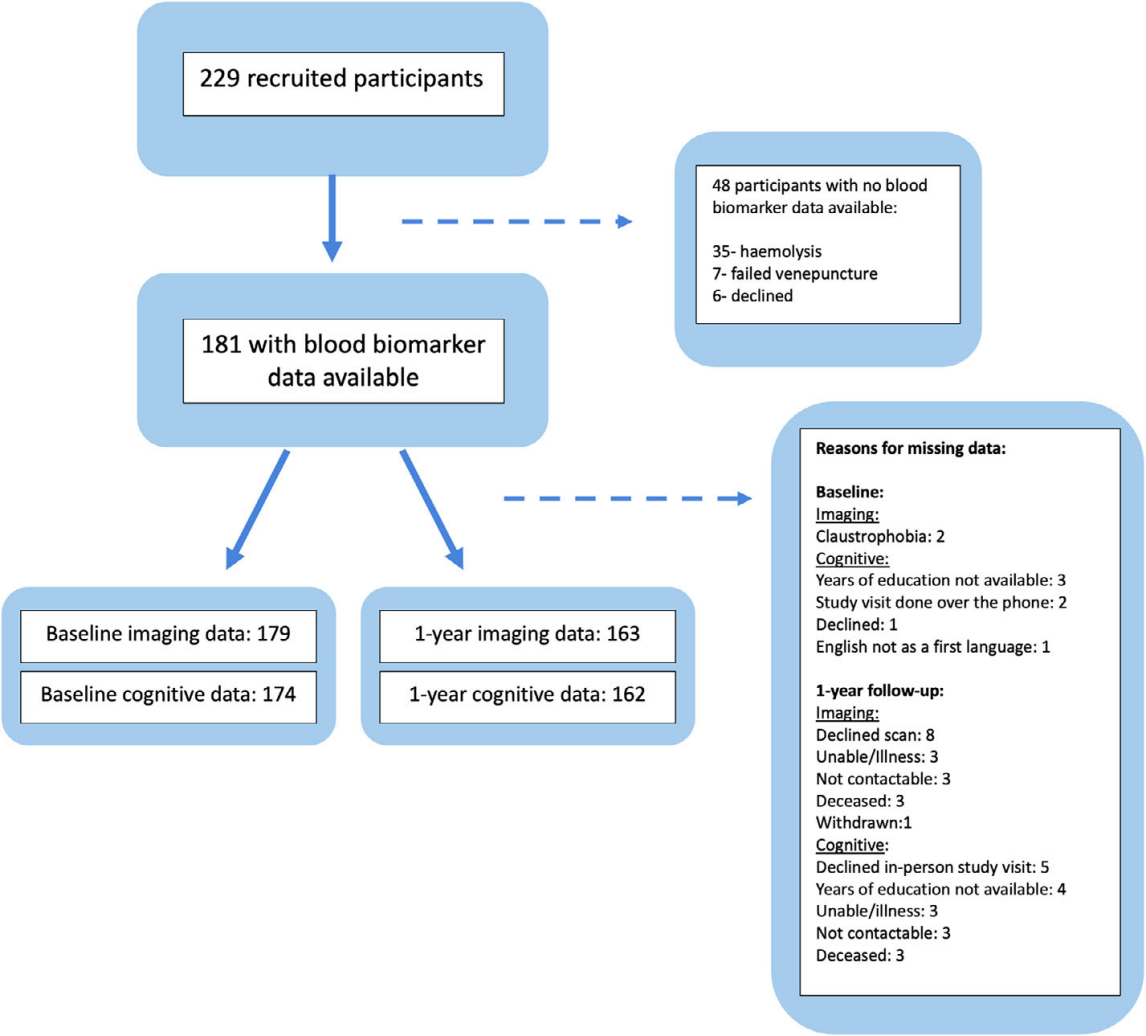
Flow diagram of study recruitment and data collection.

Of the 229 participants recruited, blood biomarker data were available for 181 (mean age: 65 years (SD:11), 31% female). We acquired baseline brain MRI (*n* = 179, including DCE‐MRI (*n* = 155) and CVR (*n* = 140)) and cognitive data (MoCA *n* = 174, TMT ratio *n* = 171) and again at 1 year (structural brain MRI *n* = 163; cognitive assessment (MoCA *n* = 162, TMT ratio *n* = 161)) (flow diagram Figures 2, S1, and S2).

Stroke subtype was lacunar in 59% of our cohort. Average WMH volume (as % ICV) was 0.93% (SD:1.1) at baseline and 1.00% (SD:1.3) at 1‐year follow‐up. Participants’ median Fazekas score at both time points was 3 (interquartile range [IQR]:3,5). The prevalence of lacunes and microbleeds at baseline was 53% and 20%, respectively, and 54% and 21% at 1‐year follow‐up. At both baseline and 1 year, the median summary SVD score was 3 (IQR:2,4). The average adjusted MoCA score in our cohort was 25 (SD:3.5) at baseline and 26 (SD:3.5) at 1 year, with 63% of patients qualifying for MCI at baseline and 46% at 1‐year follow‐up. Cohort characteristics are shown in Table 1.

**TABLE 1:** Cohort characteristics (*N *= 181)

Parameter	Overall *N* = 181
Sex, female, *n* (%)	57 (31%)
Age, years, mean (SD)	65 (11)
Stroke subtype, lacunar, *n* (%)	106 (59%)
Diabetes, yes, *n* (%)	43 (24%)
Hypertension, yes, *n* (%)	126 (70%)
Hypercholesterolemia, yes, *n* (%)	133 (73%)
Current or ex‐smoker (<1 year), *n* (%)	41 (18%)
Risk factor composite score, median (IQR)	3 (2.4)
NIHSS score, median (IQR)	2 (1.3)
Baseline NART errors, mean (SD)	17 (9.5)
Baseline WMH volume (mm^3^ as % ICV), mean (SD)	0.93 (1.1)
1‐year follow‐up WMH volume (mm^3^ as % ICV), mean (SD)^*^	1 (1.3)
Baseline summary SVD score, median (IQR)	3 (2.4)
1‐year follow‐up summary SVD score, median (IQR)^*^	3 (2.4)
Baseline Fazekas score, median (IQR)	3 (3.5)
1‐year follow‐up Fazekas score, median (IQR)^*^	3 (3.5)
Baseline, patients with ≥1 lacune, *n* (%)	94 (53%)
1‐year follow‐up, patients with ≥ 1 lacune, *n* (%)^*^	88 (54%)
Baseline, patients with ≥ 1 microbleed, *n* (%)	35 (20%)
1‐year follow‐up, patients with ≥ 1 microbleed, *n* (%)^*^	34 (21%)
Patients with new DWI‐positive lesions between baseline and 1‐year follow‐up, *n* (%)^*^	47 (26%)
Baseline MoCA score, mean (SD)	25 (3.5)
1‐year follow‐up MoCA score, mean (SD)^*^	26 (3.5)
Baseline Trail Making Test ratio (A/B), mean (SD)	2.9 (1.2)
1‐year follow‐up Trail Making Test ratio (A/B), mean (SD)^*^	2.8 (1.3)
PDGFRβ, pg/mL, mean (SD)	57,000 (23,000)
PDGF‐BB, pg/mL, mean (SD)	8900 (3300)
VCAM‐1, ng/mL, mean (SD)	2100 (760)
ICAM‐1, pg/mL, mean (SD)	200,000 (82,000)
E‐Selectin, ng/mL, mean (SD)	43 (20)
P‐Selectin, ng/mL, mean (SD)	120 (49)
Endo‐1, pg/mL, mean (SD)	3.8 (2.8)
TNF‐α, pg/mL, mean (SD)	1.2 (0.72)
vWF, mIU/mL, mean (SD)	1300 (800)
MMP‐9, pg/mL, mean (SD)	870 (440)
IL‐6, pg/mL, mean (SD)	4.2 (9.5)
VEGF, pg/mL, mean (SD)	350 (200)
PLGF, pg/mL, mean (SD)	9.6 (2.9)
PS, NAWM, ×10^4^, mean (SD)	0.16 (1.0)
PS, basal ganglia, ×10^4^, mean (SD)	0.75 (1.3)
PS, WMH, ×10^4^, mean (SD)	0.84 (1.6)
V_p_, NAWM, ×10^3^, mean (SD)	0.63 (1.8)
V_p_, basal ganglia, ×10^3^, mean (SD)	15 (3.5)
V_p_, WMH, ×x10^3^, mean (SD)	8.4 (3.7)
CVR, NAWM, %/mmHg, mean (SD)	0.043 (0.017)
CVR, basal ganglia, %/mmHg, mean (SD)	0.17 (0.053)
CVR, WMH, %/mmHg, mean (SD)	0.040 (0.043)

Abbreviations: CVR, cerebrovascular reactivity; DWI, diffusion‐weighted imaging; Endo‐1, Endothelin‐1; E‐selectin, endothelial‐selectin; ICAM‐1, intercellular adhesion molecule 1; ICV, intracranial volume; IL‐6, interleukin 6; IQR, interquartile range; MMP‐9, matrix metalloproteinase 9; MoCA, Montreal Cognitive Assessment; NART, National Adult Reading Test; NAWM, normal‐appearing white matter; NIHSS, The National Institutes of Health Stroke Scale; PDGF‐BB, platelet derived growth factor subunit B; PDGFRβ, platelet derived growth factor receptor beta; PLGF, placental growth factor; PS, permeability‐surface area product; P‐Selectin, platelet‐selectin; SVD, small vessel disease; TNF‐α, tumor necrosis factor alpha; VCAM‐1, vascular cell adhesion molecule 1; VEGF, vascular endothelial growth factor; V_p_, blood plasma volume fraction; vWF, von Willebrand Factor; WMH, white matter hyperintensity.

^*^ Longitudinal 1‐year imaging data were available for 163 participants and cognitive data for 162 participants.

Compared to participants without biomarker data, those with available biomarker data were younger (mean age: 65 years (SD:11) vs. 68 (SD:10)) and had more extensive WMH lesions at baseline and 1 year (mean WMH volume: 0.96% (SD:1.2), and 1.00% (SD:1.3), respectively, vs. 0.85% (SD:0.93) and 0.99% (SD:1.2)). They also had fewer lacunes at baseline (median: 1 (IQR:1,3) vs. 2 (IQR:1,3)) and 1 year (median: 1 (IQR:0,3) vs. 2 (IQR:1,4)) as well as microbleeds, both at baseline (median: 0 (IQR:0,0) vs. 1 (IQR:1,1)) and 1 year (median: 0 (IQR:0,0) vs. 1 (IQR:1,1)) (Table S1).

Of those with available biomarker data, participants who attended the 1‐year follow‐up with brain MRI (*n* = 163 out of 181) were of comparable age, had similar WMH volumes and summary SVD and Fazekas scores at baseline, had more lacunes (2.1 (SD:3.3) vs. 1.4 (SD:1.9)) and microbleeds (1.4 (SD:5.6) vs. 0.71 (SD:2.4)) on average, and comparable cognitive assessment scores to those who did not attend the 1‐year follow‐up (Table S2).

Compared to cortical stroke patients, lacunar stroke patients were more likely to have diabetes (28% vs.18%), hypertension (74% vs. 65%), hypercholesterolemia (79% vs. 66%), be current smokers (15% vs. 9%), have higher WMH volumes (1% (SD:1.2) vs. 0.84% (SD:1.1)), have a higher SVD summary score (3.1 (SD:1.3) vs. 2.5 (SD:1.3)), have more lacunes (2.8 (SD:3.8) vs. 0.95 (SD:1.7)) and microbleeds (1.9 (SD:6.6) vs. 0.58 (SD:2.7)) at baseline, and at 1 year (3.0 (SD:3.9) vs. 0.98 (SD:1.8), and 2.8 (SD:8.3) vs. 0.51 (SD:2.6), respectively), and more DWI‐positive lesions detected throughout 1 year (0.73 (SD:1.4) vs. 0.28 (SD:0.65)). Cohort characteristics of lacunar stroke patients compared to cortical stroke patients with blood biomarker data can be found in Table S3.

Serology was performed outside the acute stroke phase, and no significant correlation was found between individual biomarkers and the time elapsed from stroke onset to serology (mean: 59.56 days (SD:21.65)) (Table S4).

### 3.1 Imaging outcomes

#### 3.1.1 WMH: Cross‐sectional and longitudinal WMH volumes and baseline Fazekas score

In linear mixed effects models adjusted for age, sex, vascular risk factors, stroke subtype, and baseline NIHSS, we detected trends wherein higher circulating levels of PDGF‐BB (*β* = 0.11, 95% confidence interval [CI]: –0.04, 0.26), MMP‐9 (*β* = 0.09, 95% CI: –0.06, 0.24), and Endo‐1 (*β* = 0.06, 95% CI: –0.09, 0.21) showed possible associations with increasing WMH volumes longitudinally; similar patterns were evident cross‐sectionally (Figure S3 and Table S5). In ordinal logistic regression analyses featuring Fazekas scores at baseline, PDGF‐BB (odds ratio [OR]:1.15, 95% CI:0.86, 1.55), E‐Selectin (OR:1.12, 95% CI:0.84, 1.51), and ICAM‐1 (OR:1.09, 95% CI:0.82, 1.50) showed potential associations with higher WMH burden cross‐sectionally (Figure S4 and Table S6).

### 3.2 Prevalent lacunes and microbleeds at baseline

In logistic regression models adjusted for age, sex, vascular risk factors, and stroke subtype, PDGFRβ and Endo‐1 were associated with decreased odds of lacunes (OR per 1 SD increase in PDGFRβ = 0.71, 95% CI:0.50, 0.99; OR per 1 SD increase in Endo‐1 = 0.71, 95% CI:0.49, 0.99). ICAM‐1 (OR per 1 SD increase in ICAM‐1 = 1.26, 95% CI:0.91, 1.77), E‐Selectin (OR per 1 SD increase in E‐Selectin = 1.15, 95% CI:0.82, 1.67) and vWF (OR per 1 SD increase in vWF = 1.14, 95% CI:0.81, 1.64), showed possible associations with lacunes at baseline, while VEGF (OR per 1 SD increase in VEGF = 1.27, 95% CI:0.86, 1.86) showed potential links to microbleeds (Figure S5 and Table S7).

### 3.3 Incident DWI‐positive lesions longitudinally

**FIGURE 3:**
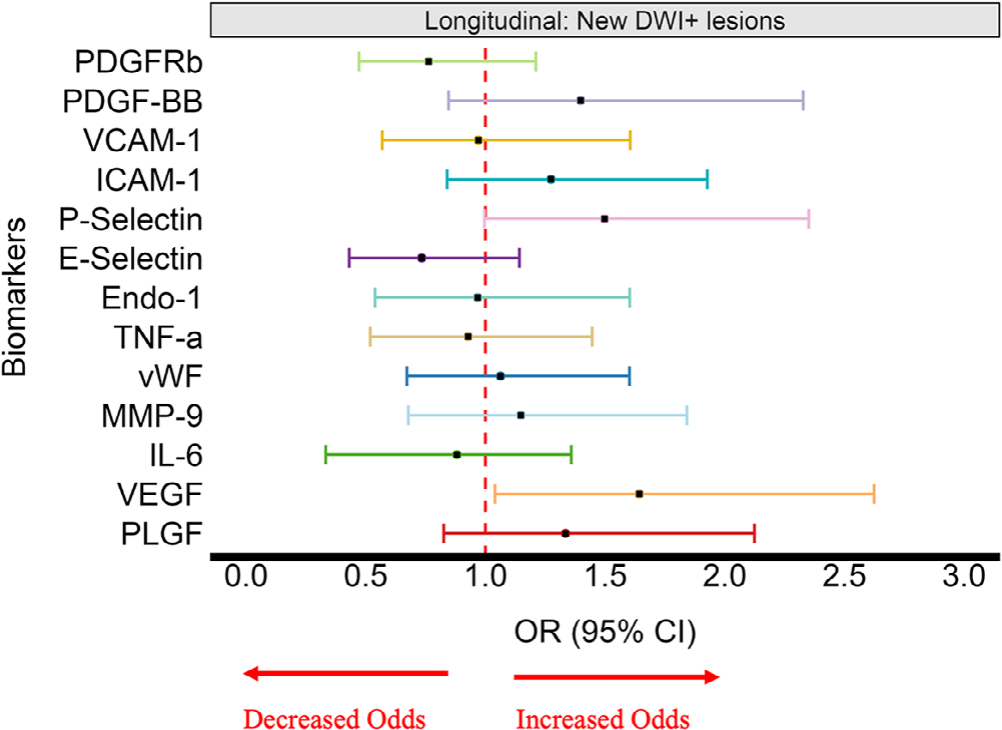
Blood biomarkers and newly appearing incident lesions (DWI‐positive) over 1 year. Logistic regression results for the association of distinct standardized blood biomarkers to the prevalence of DWI‐positive incident lesions identified throughout the 1‐year follow‐up period after adjusting for age, sex, vascular risk factors, stroke subtype, and baseline NIHSS. CI, confidence interval; DWI, diffusion‐weighted imaging; E‐Selectin, endothelial‐selectin; Endo‐1, Endothelin‐1; ICAM‐1, intercellular adhesion molecule 1; IL‐6, interleukin 6; MMP‐9, matrix metalloproteinase 9; NIHSS, The National Institutes of Health Stroke Scale; P‐Selectin, platelet‐selectin; PDGF‐BB, platelet derived growth factor subunit B; PDGFRβ, platelet derived growth factor receptor beta; PlGF, placental growth factor; TNF‐α, tumor necrosis factor alpha; VCAM‐1, vascular cell adhesion molecule 1; VEGF, vascular endothelial growth factor; vWF, von Willebrand Factor.

Results from logistic regression adjusted for age, sex, vascular risk factors, and stroke subtype, revealed a significant association between higher VEGF concentrations and a 1.64‐fold increased odds of incident DWI‐positive lesions identified between baseline and 1‐year follow up (OR per 1 SD increase in VEGF = 1.64; 95% CI:1.04, 2.62). We also observed possible trends between higher levels of P‐Selectin (OR per 1 SD increase in P‐Selectin = 1.49; 95% CI:0.99, 2.35), PDGF‐BB (OR per 1 SD increase in PDGF‐BB = 1.40; 95% CI:0.85, 2.32), PlGF (OR per 1 SD increase in PlGF = 1.34; 95% CI:0.83, 2.12), and ICAM‐1 (OR per 1 SD increase in ICAM‐1 = 1.27; 95% CI:0.84, 1.93), and increased odds of incident DWI‐positive lesion detection (Figure 3 and Table S8).

### 3.4 Summary SVD scores at baseline

Results from ordinal regression adjusted for age, sex, vascular risk factors, and stroke subtype, indicate a possible trend between elevated concentrations of ICAM‐1 (OR per 1 SD increase in ICAM‐1 = 1.18; 95% CI:0.89, 1.57), PDGF‐BB (OR per 1 SD increase in PDGF‐BB = 1.17; 95% CI:0.88, 1.57), and E‐selectin (OR per 1 SD increase in E‐Selectin = 1.11; 95% CI:0.86, 1.47) and summary SVD burden observed on MRI, encompassing WMH, PVS, lacunes, and microbleeds (Figure S6 and Table S9).

### 3.5 BBB permeability at baseline

**FIGURE 4:**
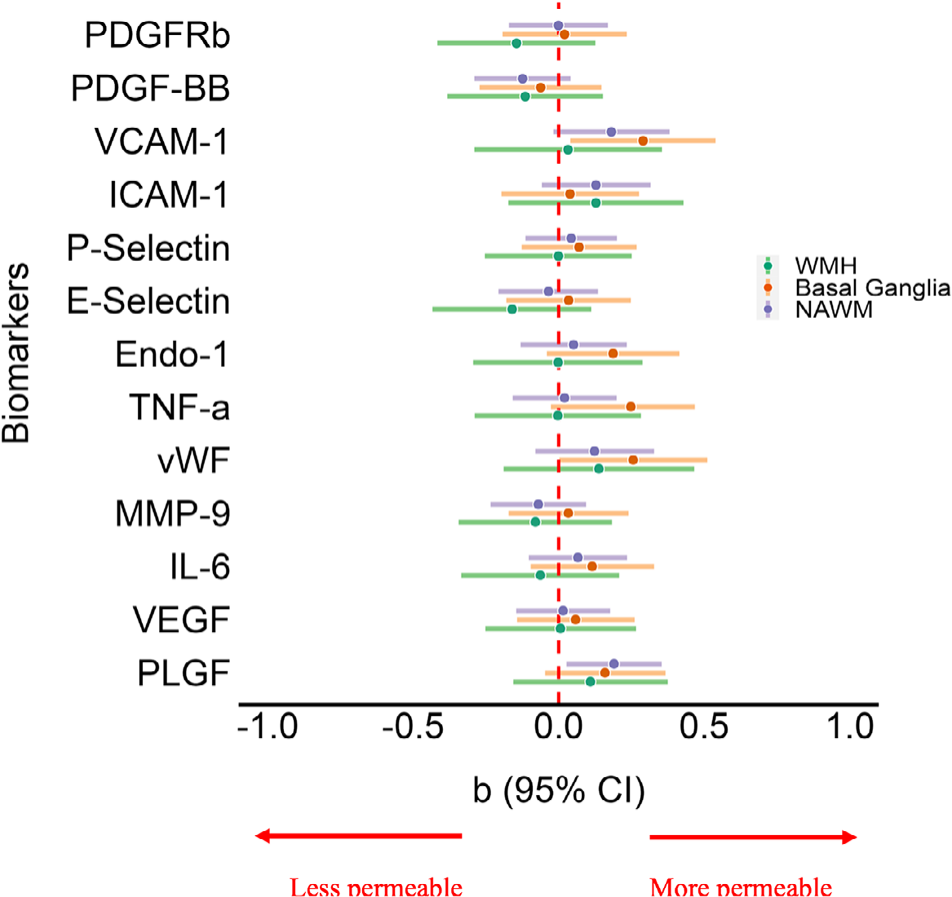
Blood biomarkers and BBB permeability (PS) from DCE‐MRI. Linear regression results for the association of distinct standardized blood biomarkers to baseline DCE‐MRI parameter PS (×10^4^) after adjusting for age, sex, vascular risk factors, and baseline WMH volume as % ICV. BBB, blood‐brain barrier; CI, confidence interval; DCE‐MRI, dynamic contrast‐enhanced magnetic resonance imaging; E‐Selectin, endothelial‐selectin; Endo‐1, Endothelin‐1; ICAM‐1, intercellular adhesion molecule 1; ICV, intracranial volume; IL‐6, Interleukin 6; MMP‐9, matrix metalloproteinase 9; P‐Selectin, platelet‐selectin; PDGF‐BB, platelet derived growth factor subunit B; PDGFRβ, platelet derived growth factor receptor beta; PlGF, placental growth factor; PS, permeability‐surface area product; TNF‐α, tumor necrosis factor alpha; VCAM‐1, vascular cell adhesion molecule 1; VEGF, vascular endothelial growth factor; vWF, von Willebrand factor; WMH, white matter hyperintensity.

In cross‐sectional linear regression models adjusted for age, sex, vascular risk factors, and baseline WMH volume, we observed possible associations between higher concentrations of VCAM‐1, ICAM‐1, vWF, TNF‐α, PlGF, and Endo‐1 and greater BBB permeability (PS), with some variation dependent on tissue type (NAWM, basal ganglia, and WMH) (Figure 4 and Table S10). For instance, in NAWM, levels of PlGF were significantly associated with increased BBB permeability (*β* = 0.19, 95% CI: 0.03, 0.35), while in the basal ganglia, VCAM‐1 (*β* = 0.29, 95% CI:0.04, 0.54), TNF‐α (*β* = 0.25, 95% CI:0.03, 0.47), and vWF (β = 0.26, 95% CI:0.00, 0.51), levels were significantly associated with increased BBB permeability. Interestingly, we observed a possible association between higher serum levels of PDGF‐BB and reduced BBB permeability in NAWM (*β* = –0.12, 95% CI: –0.29, 0.04), the basal ganglia (*β* = –0.06, 95% CI: –0.27, 0.15), and WMH (*β* = –0.12, 95% CI: –0.38, 0.15). Increased ICAM‐1 and PDGF‐BB concentrations showed possible associations with increased vascular density through V_p_ quantification (Table S11).

### 3.6 Cerebrovascular reactivity at baseline

**FIGURE 5:**
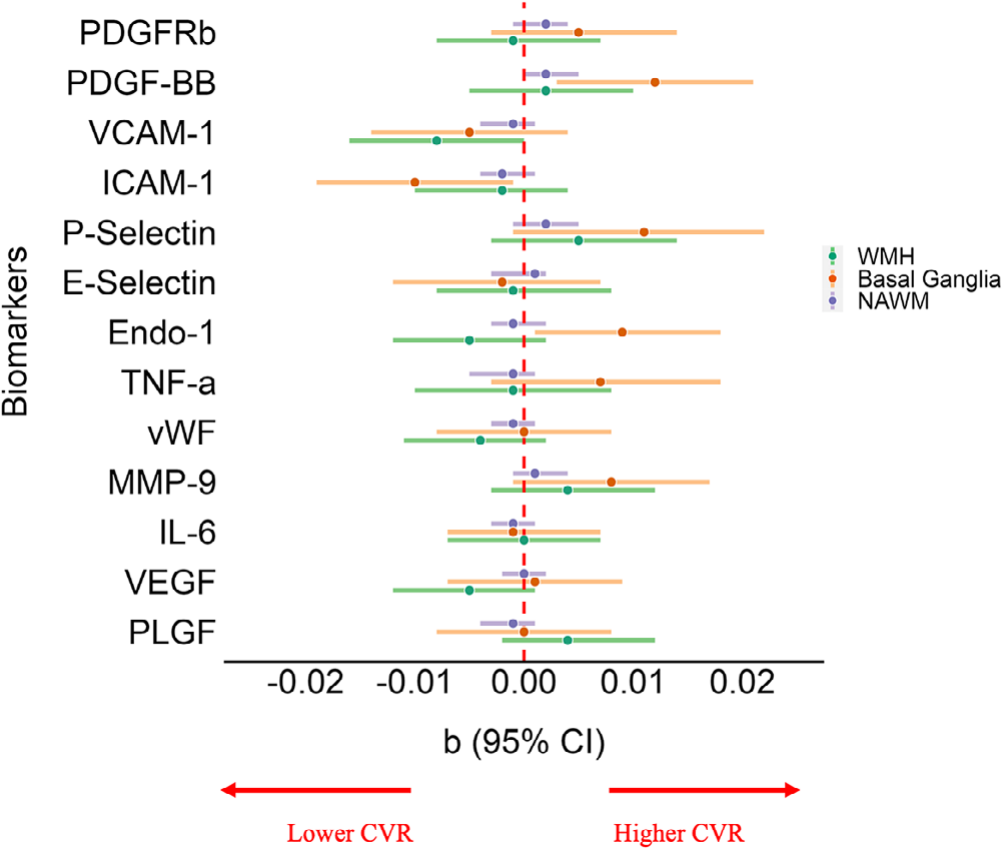
Blood biomarkers and CVR. Linear regression results for the association of distinct standardized blood biomarkers to baseline DCE‐CVR in NAWM, the basal ganglia and WMH after adjusting for age, sex, vascular risk factors, and baseline WMH volume as % ICV. CI, confidence interval; CVR, cerebrovascular reactivity; E‐selectin, endothelial‐selectin; Endo‐1, Endothelin‐1; ICAM‐1, intercellular adhesion molecule 1; ICV, intracranial volume; IL‐6, interleukin 6; MMP‐9, matrix metalloproteinase 9; NAWM, normal appearing white matter; P‐Selectin, platelet‐selectin; PDGF‐BB, platelet derived growth factor subunit B; PDGFRβ, platelet derived growth factor receptor beta; PlGF, placental growth factor; TNF‐α, tumor necrosis factor alpha; VCAM‐1, vascular cell adhesion molecule 1; VEGF, vascular endothelial growth factor; vWF, von Willebrand Factor; WMH, white matter hyperintensity.

Higher circulating levels of ICAM‐1 were significantly associated with lower CVR at baseline, particularly in the basal ganglia (*β* = –0.01, 95% CI: –0.02, –0.001) after adjusting for age, sex, vascular risk factors, and baseline WMH volume (Figure 5 and Table S12). We also observed a possible association between higher levels of ICAM‐1 and lower CVR in NAWM (*β* = ‐0.002, 95% CI:‐0.004, 0.001) and WMH (*β* = ‐0.002, 95% CI:‐0.010, 0.004), as well as increased VCAM‐1 levels and lower CVR in all tissue types (NAWM: *β* = ‐0.001, 95% CI:‐0.004, 0.001; basal ganglia: *β* = ‐0.01, 95% CI:‐0.01, 0.004; WMH: (*β* = ‐0.01, 95% CI:‐0.02, ‐0.001).

Conversely, higher levels of PDGF‐BB and Endo‐1 were significantly associated with higher CVR, particularly in the basal ganglia (*β* = 0.01, 95% CI:0.003, 0.02; *β* = 0.01, 95% CI:0.001, 0.02, respectively).

### 3.7 Cognitive Outcomes

#### 3.7.1 MoCA scores and mild cognitive impairment

**FIGURE 6:**
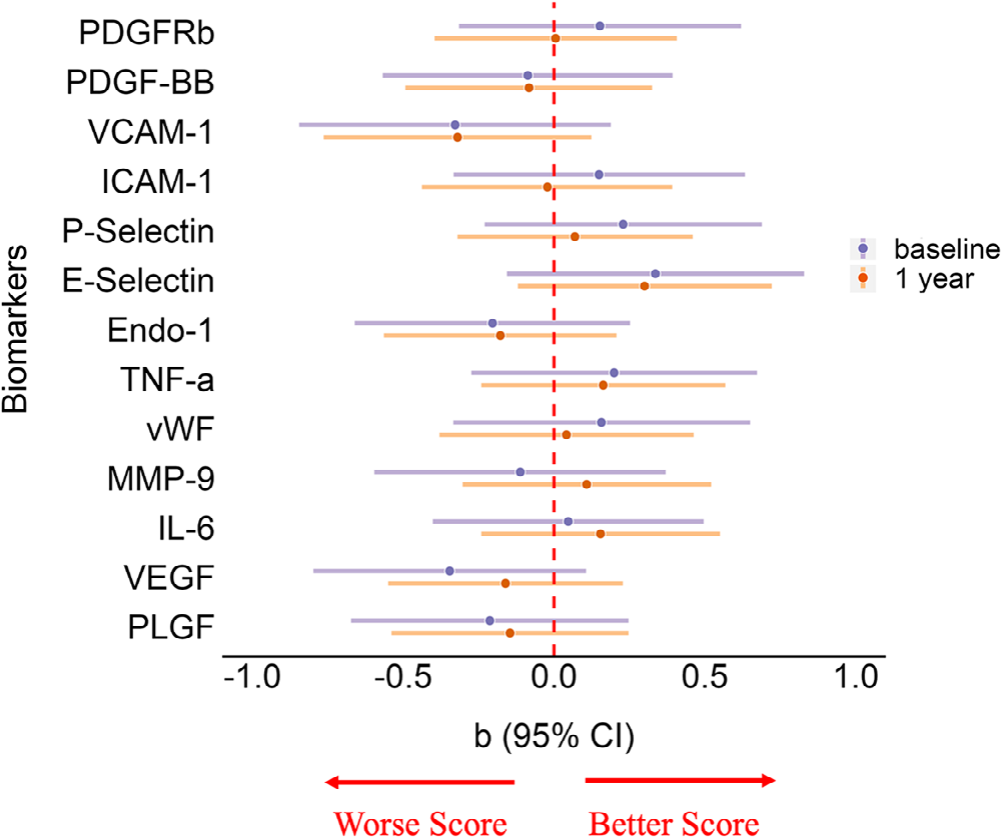
Blood biomarkers and MoCA score. Baseline cross‐sectional linear regression and longitudinal linear mixed effects model results for the association of distinct standardized blood biomarkers and MoCA score after adjusting for age, sex, vascular risk factors, premorbid intelligence, and baseline WMH volume as % ICV. ICV, intracranial volume; CI, confidence interval; E‐Selectin, endothelial‐selectin; Endo‐1, Endothelin‐1; IL‐6, interleukin 6; MMP‐9, matrix metalloproteinase 9; MoCA, Montreal Cognitive Assessment; PDGF‐BB, platelet derived growth factor subunit B; PDGFRβ, platelet derived growth factor receptor beta; ICAM‐1, intercellular adhesion molecule 1; P‐Selectin, platelet‐selectin; PlGF, placental growth factor; TNF‐α, tumor necrosis factor alpha; VCAM‐1, vascular cell adhesion molecule 1; VEGF, vascular endothelial growth factor; vWF, von Willebrand Factor; WMH, white matter hyperintensity.

Longitudinal linear mixed effects models adjusted for age, sex, vascular risk factors, premorbid intelligence, and baseline WMH volume suggest a plausible association between higher concentrations of VCAM‐1 (*β* = ‐0.32, 95% CI:‐0.76, 0.12), Endo 1 (*β* = ‐0.18, 95% CI:‐0.56, 0.21), VEGF (*β* = ‐0.16, 95% CI:‐0.55, 0.23), and PlGF (*β* = ‐0.15, 95% CI:‐0.54, 0.25) and lower MoCA scores. Cross‐sectional analyses examining baseline MoCA scores showed comparable results (Figure 6 and Table S13).

At baseline, 36% of patients had normal cognition, while 63% qualified for MCI. At 1‐year follow‐up, 53% of patients had normal cognition and 46% qualified for MCI or dementia. After adjusting for age, sex, vascular risk factors, premorbid intelligence, and baseline WMH volume, we found a significant relationship between higher concentrations of P‐Selectin (OR per 1 SD increase in P‐Selectin = 1.65; 95% CI:1.05, 2.72) and increased risk of MCI at 1 year as well as higher levels of IL‐6 (OR per 1 SD increase in IL‐6 = 0.39; 95% CI:0.07, 0.94) and a reduced risk of MCI at 1 year.

We also observed potential associations between increased risk of MCI prevalence and higher concentrations of PDGF‐BB (1 year: OR per 1 SD increase in PDGF‐BB = 1.29; 95% CI:0.90, 1.90), VCAM‐1 (baseline: OR per 1 SD increase in VCAM‐1 = 1.48; 95% CI:0.96, 2.37; 1 year: OR = 1.50; 95% CI:0.98, 2.40), ICAM‐1 (1 year: OR per 1 SD increase in ICAM‐1 = 1.34; 95% CI:0.90, 2.04), vWF (baseline: OR per 1 SD increase in vWF = 1.37; 95% CI:0.90, 2.26; 1 year: OR = 1.25; 95% CI:0.84, 1.94), MMP‐9 (baseline: OR per 1 SD increase in MMP‐9 = 1.46; 95% CI:0.98, 2.28), VEGF (baseline: OR per 1 SD increase in VEGF = 1.31; 95% CI:0.93, 1.89), and PlGF (baseline: OR per 1 SD increase in PlGF = 1.14; 95% CI:0.80, 1.63) (Figure S7 and Table S14).

### 3.8 Executive function: TMT ratio (B/A)

We examined possible associations between executive function, as measured by the TMT (B/A) ratio, and circulating biomarkers, both cross‐sectionally at baseline, and longitudinally using linear mixed‐effects models. After adjusting for age, sex, vascular risk factors, premorbid intelligence, and baseline WMH volume, we observed possible associations between higher levels of Endo‐1 (baseline: *β* = 0.11, 95% CI:–0.06, 0.28; longitudinally: *β* = 0.14, 95% CI:–0.01, 0.30), TNF‐α (baseline: *β* = 0.14, 95% CI:–0.04, 0.32; longitudinally: *β* = 0.08, 95% CI:–0.07, 0.23), vWF (baseline: *β* = 0.13, 95% CI:–0.05, 0.32; longitudinally: *β* = 0.19, 95% CI:0.03, 0.35), and IL‐6 (baseline: *β* = 0.11, 95% CI:–0.06, 0.27; longitudinally: *β* = 0.05, 95% CI:–0.10, 0.20), and worse executive function (Figure S8 and Table S15). Results also suggested potential associations between higher levels of VEGF and PlGF and better executive function, both at baseline (*β* = –0.16, 95% CI:–0.33, 0.01 and *β* = –0.19, 95% CI:–0.36, –0.02, respectively) and longitudinally (*β* = –0.15, 95% CI:–0.30, 0.01 and *β* = –0.10, 95% CI:–0.25, 0.05, respectively).

## 4 DISCUSSION

We performed cross‐sectional and 1‐year longitudinal adjusted analyses relating 13 blood biomarkers of endothelial, vascular, and pericyte function, to MRI markers of SVD, including WMH, lacunes, microbleeds, summary SVD score, BBB permeability, and CVR, as well as cognition and MCI prevalence in a minor stroke cohort enriched for SVD (Figure S9).

We found a novel significant association between circulating VEGF levels and increased odds of incident DWI‐positive lesions throughout the 1‐year follow‐up period. VEGF and PlGF are angiogenic factors implicated in endothelial cell proliferation and survival and microvascular permeability.^27^ There is evidence suggesting that angiogenic signaling alterations and subsequent blood flow changes are key features of endothelial dysfunction in ageing and could contribute to inflammation and BBB permeability, possibly by uncoupling endothelial cell junctions.^28^, ^29^ Results from recent studies are inconsistent. In the Framingham offspring cohort, no associations were reported between VEGF and WMH burden, microbleeds, or lacunes.^30^ Other studies report links between VEGF and WMH burden, lacunes, and greater global SVD disease burden, both in acute ischemic stroke patients and in cognitively‐unimpaired elderly community dwellers.^31^, ^32^ Results from the ongoing MarkVCID consortium demonstrated strong associations between PlGF and WMH burden and cognitive impairment, although associations with executive function were modest.^33^ In our study, VEGF and PlGF concentrations showed possible associations with lower MoCA scores, and increased odds of MCI at baseline and incident DWI‐positive lesions, however, we found no overt relationship with WMH burden, and, interestingly, both VEGF and PlGF levels showed potential positive associations with better executive functioning. PlGF also showed an association with higher BBB permeability in NAWM whereas VEGF was possibly linked to the presence of microbleeds.

We found significant associations between higher concentrations of VCAM‐1 and increased BBB permeability in the basal ganglia, ICAM‐1 and lower CVR, and P‐Selectin and an increased risk of MCI at 1 year. Cell adhesion molecules and selectins, VCAM‐1, ICAM‐1, P‐Selectin, and E‐Selectin, promote the recruitment, rolling, adhesion, and trans‐endothelial migration of leukocytes through vascular walls. They are constitutively expressed at low levels in the brain vasculature in a homeostatic state and their increased expression can indicate endothelial activation.^34^ We observed trends between higher ICAM‐1 levels and lacune and incident DWI‐positive lesion prevalence as well as higher summary SVD score, BBB permeability and higher likelihood of MCI at one year. Our results support those reported in the Framingham cohort study, where ICAM‐1 levels were associated with higher WMH volume and silent brain infarcts.^30^ In our study, increasing VCAM‐1 concentrations showed possible associations with BBB breakdown and worse cognition, yet a lower WMH burden. Previous studies report conflicting results.^6^ VCAM‐1 concentrations were elevated in lacunar stroke patients compared to hypertensive and healthy controls, and higher concentrations correlated with poorer cognition and higher WMH burden.^35^, ^36^ The Rotterdam cohort study reported no relationship between ICAM‐1 or VCAM‐1 levels and dementia diagnosis.^37^ In our study, E‐Selectin levels showed potential associations with worse WMH burden, higher summary SVD score, and higher baseline lacune and MCI prevalence. P‐Selectin showed possible associations with DWI‐positive lesion prevalence and MCI incidence, though not WMH burden. While some studies report higher circulating levels of selectins in SVD compared to controls, others report no differences.^38^, ^39^ In a 10‐month follow‐up study, E‐Selectin was associated with SVD progression.^40^ Likewise, those with SVD radiological disease progression over 2 years had a higher compound endothelial activation score, including P‐Selectin.^41^

We found possible associations between biomarkers of pericyte function and cognitive impairment and SVD MRI markers. Brain pericytes rely on PDGF‐BB and PDGFRβ signaling to maintain the BBB.^42^, ^43^ PDGF‐BB is secreted by the endothelium and recruits pericytes to endothelial cells, while its receptor, PDGFRβ, is enriched in pericytes. Increased soluble PDGFRβ secretion is thought to indicate pericyte injury and has been proposed as a BBB breakdown biomarker.^44^, ^45^, ^46^, ^47^ Increased cerebrospinal fluid (CSF) concentrations of PDGFRβ have been implicated in MCI and BBB dysfunction in SVD and Alzheimer's Disease (AD).^45^, ^48^, ^49^ In our study, PDGF‐BB trended toward an association with higher WMH burden, increased incident DWI‐positive lesions, microbleeds, higher global SVD burden, lower cognition, and a higher risk of MCI. Interestingly, circulating levels of PDGFRβ showed possible associations with lower WMH burden, lower MCI risk, and higher MoCA scores. An interaction between these molecules throughout different disease stages, perhaps even in a compensatory capacity through the attempt to increase pericyte recruitment as a response to pericyte loss, is conceivable. In animal and in vitro investigations, PDGF‐BB was overexpressed in vascular sections with high wall‐shear stress and was implicated in inciting an inflammatory response and smooth muscle cell dedifferentiation via PDGFRβ signaling mechanisms.^50^, ^51^ Alternatively, it remains possible that quantifying these markers in the circulating blood does not serve as a reliable proxy for brain pericyte (dys)function; further examination, ideally with CSF samples, is warranted.

We observed a significant association between vWF and BBB permeability, and possible associations with worse cognition, increased odds of lacunes at baseline, and lower WMH burden. vWF secretion can be incited by inflammation and is involved in thrombus formation and platelet adhesion. In wild‐type mice, vWF increased BBB permeability by inhibiting the expression of tight junction, claudin‐5.^52^ Although several studies report higher circulating levels of vWF in recent lacunar stroke patients compared to controls, others suggest these levels normalize past the acute‐stroke stage or are comparable to patients of non‐lacunar ischemic stroke.^39^, ^53^, ^54^ One study found no relationship between vWF levels and WMH or lacunes on CT; however, only a small proportion of patients presented with lacunar stroke (12%), and WMH burden was low.^31^ There are possible explanations for the inverse relationship between WMH burden and vWF. While high levels of vWF are associated with vascular inflammation and stroke, it has also been implicated in the promotion of endothelial integrity^52^, ^55^; lower vWF levels have been associated with increased PVS burden.^56^ Some studies suggest vWF can be differentiated between an active and latent form, which may play diverging roles in hemostasis.^57^ Regulating endothelial cell‐mediated vWF secretion could serve as a potential therapeutic target, though further examination into its role in modulating endothelial and BBB integrity, particularly as it relates to an acute versus chronic response to injury or disease, is required.

The strengths of this study lie in its longitudinal aspect and the comprehensive range of disease‐related markers and outcomes, which are rarely available in larger cohorts. These include vascular function MRI markers, such as CVR and BBB permeability, alongside multiple radiological indicators of disease and global SVD burden quantification to effectively capture the evolving radiological landscape of SVD. Moreover, including cortical stroke patients with varying levels of SVD, alongside conducting serology outside the acute stroke phase, serves to minimize potential confounding effects related to acute ischemic injury. We have also accounted for clinical and vascular risk factor data within our models.

Our study has several limitations. Our relatively small sample size was predominantly of European descent, limiting the generalizability of our findings. Although our study is longitudinal, a 1‐year period might not be sufficient to capture relationships between biomarkers and disease progression; additionally, serology was only performed at baseline, and we cannot make inferences about the stability of analytes examined over time. We adjusted for stroke subtype and included cortical strokes as a control group since they receive the same secondary prevention drugs as do lacunar stroke patients. However, ways in which large‐artery atherosclerotic disease mechanisms may influence vascular function biomarkers despite representing distinct pathological processes, remains unclear. Because all patients received routine secondary prevention medication, we did not include medication use in our models. Although effects will likely be similar across the population, how they may affect biomarkers is unknown. Participants were not excluded for concurrent infection, malignancy, or rheumatological disease, which might affect the interpretation of our results. We relied on ELISA for biomarker quantification, acknowledging that different serology techniques could produce different results with varying levels of accuracy. Notably, our approach does not always facilitate the differentiation of diverse biomarker isoforms, despite the potential for these to have opposing roles.^58^ We primarily used serum samples for our analyses, with plasma substituting less than 4% of the samples; although testing and using separate dilutions can mitigate some variability, inherent differences between the two matrices may affect results. Importantly, the extent to which these biomarkers accurately reflect dysfunction confined to the brain rather than systemic dysfunction, and the degree to which endothelial/pericyte dysfunction in SVD is indeed confined to the brain, remains unknown. Last, this study was exploratory in nature, and replication in external samples will be necessary to derive more robust conclusions.

A major challenge impeding the advancement of therapeutics in SVD arises from the absence of elucidative and specific biomarkers associated with disease. Establishing disease‐specific biomarkers could facilitate efforts to uncover pathophysiology and develop potential therapeutic interventions and evaluate their effectiveness. These novel findings from a human stroke population enriched for SVD can help identify key candidate biomarkers for further investigation in SVD research.

In clinical settings, biomarkers could serve as accessible and specific diagnostic and prognostic tools. We found a novel association between circulating VEGF levels and increased incident DWI‐positive lesions throughout the first year post‐stroke as well as associations between higher levels of P‐Selectin and increased risk for MCI at 1 year and ICAM‐1 and lower CVR. If validated in larger populations, these findings could have major clinical implications in stroke clinics, emergency departments, and clinical trial design by facilitating the risk‐stratification of patients who may be at a higher risk of recurrent stroke and rapid‐progression SVD.

## CONFLICT OF INTEREST STATEMENT

Alasdair G. Morgan, Cameron Manning, and Michael S. Stringer were part‐funded by Siemens Healthineers, administered by the University of Edinburgh. The other authors report no conflicts. Author disclosures are available in the Supporting Information.

## CONSENT STATEMENT

All human subjects provided informed consent.

## Supporting information



Supporting Information

Supporting Information

Supporting Information
