# Supplementary material for: Blood biomarkers of vascular dysfunction in small vessel disease progression: Insights from a longitudinal neuroimaging study
Source: Alzheimers Dement. 2025 Apr 25;21(4):e70152. doi: 10.1002/alz.70152 (PMC12022501; doi:10.1002/alz.70152)
Supplement: Supplementary file 1 — Supporting Information [file ALZ-21-e70152-s003.docx]

# Supplemental Materials

# Tables

**Table S1. Cohort characteristics of patients with available blood biomarker data compared to patients without.**

|  | | | |
| --- | --- | --- | --- |
|  | **No (N=48)** | **Yes (N=181)** | **Overall (N=229)** |
| **Sex, female, n (%)** | 21 (43 %) | 57 (31 %) | 78 (34 %) |
| **Age, years, mean (SD)** | 68 (± 10) | 65 (± 11) | 66 (± 11) |
| **Stroke subtype, lacunar, n (%)** | 25 (51 %) | 106 (59 %) | 131 (57 %) |
| **Diabetes, yes, n (%)** | 7 (14 %) | 43 (24 %) | 50 (22 %) |
| **Hypertension, yes, n (%)** | 31 (63 %) | 126 (70 %) | 157 (68 %) |
| **Hypercholesterolaemia, yes, n (%)** | 38 (78 %) | 133 (73 %) | 171 (74 %) |
| **Current smoker or ex-smoker < 1 year ago, n (%)** | 10 (20 %) | 31 (17 %) | 41 (18 %) |
| **Risk factor score, mean (SD)** | 2.8 (± 0.97) | 2.8 (± 0.96) | 2.8 (± 0.96) |
| **Baseline NIHSS, mean (SD)** | 2.1 (± 1.1) | 2.4 (± 1.4) | 2.3 (± 1.4) |
| **Baseline NART score, number of errors, mean (SD)** | 17 (± 9.7) | 18 (± 9.5) | 17 (± 9.5) |
| **Baseline WMH volume (mm^3^ as % ICV), mean (SD)** | 0.85 (± 0.93) | 0.96 (± 1.2) | 0.93 (± 1.1) |
| **1-year follow-up WMH volume (mm^3^ as % ICV), mean (SD) ^*^** | 0.99 (± 1.2) | 1.0 (± 1.3) | 1.0 (± 1.3) |
| **Change in WMH volume between baseline and 1-year follow-up (mm^3^ as % ICV), mean (SD)^*^** | 0.086 (± 0.29) | 0.068 (± 0.20) | 0.071 (± 0.22) |
| **Baseline SVD composite score, mean (SD)** | 2.8 (± 1.3) | 2.8 (± 1.4) | 2.8 (± 1.3) |
| **1-year follow-up SVD composite score, mean (SD)^*^** | 2.7 (± 1.2) | 2.8 (± 1.3) | 2.8 (± 1.3) |
| **Baseline Fazekas score, mean (SD)** | 4.1 (± 1.4) | 4.0 (± 1.6) | 4.1 (± 1.6) |
| **1-year follow-up Fazekas score, mean (SD)^*^** | 4.2 (± 1.5) | 4.0 (± 1.7) | 4.1 (± 1.6) |
| **Lacunes at baseline, number, median (IQR)** | 2.0 (1,3) | 1.0 (1,3) | 2.0 (1,4) |
| **Lacunes at 1-year follow-up, number, median (IQR)^*^** | 2.0 (1,4) | 1.0 (0,3) | 2.0 (1,4) |
| **Microbleeds at baseline, number, median (IQR)** | 1.0 (1,1) | 0.0 (0,0) | 1.0 (1,1) |
| **Microbleeds at 1-year follow-up, number, median (IQR)^*^** | 1.0 (1,1) | 0.0 (0,0) | 1.0 (1,1) |
| **New DWI-positive lesions between baseline and 1-year follow-up detected, number, mean (SD)^*^** | 0.37 (± 0.91) | 0.54 (± 1.2) | 0.50 (± 1.1) |
| **MoCA score at baseline, mean (SD)** | 25 (± 3.2) | 25 (± 3.5) | 25 (± 3.5) |
| **MoCA score at 1-year follow-up, mean (SD)^*^** | 26 (± 3.2) | 26 (± 3.6) | 26 (± 3.5) |
| **Baseline Trail Making Test ratio (A/B), mean (SD)** | 3 (1.6) | 2.9 (1.2) | 2.9 (1.3) |
| **1-year follow-up Trail Making Test ratio (A/B), mean (SD)^*^** | 2.7 (1.6) | 2.8 (1.3) | 2.8 (1.4) |
| **PS, NAWM, x10^4^, mean (SD)** | 0.23 (0.98) | 0.16 (1.0) | 0.17 (1.0) |
| **PS, basal ganglia, x10^4^, mean (SD)** | 0.85 (1.2) | 0.75 (1.3) | 0.77 (1.3) |
| **PS, WMH, x10^4^, mean (SD)** | 0.99 (1.7) | 0.84 (1.6) | 0.87 (1.6) |
| **V_p_, NAWM, x10^3^, mean (SD)** | 7.0 (2.0) | 0.63 (1.8) | 6.4 (1.9) |
| **V_p_, basal ganglia, x10^3^, mean (SD)** | 16 (3.7) | 15 (3.5) | 15 (3.6) |
| **V_p_, WMH, x10^3^, mean (SD)** | 9.4 (4.0) | 8.4 (3.7) | 8.6 (3.8) |
| **CVR, NAWM, % /mmHg, mean (SD)** | 0.041 (0.014) | 0.043 (0.017) | 0.043 (0.016) |
| **CVR, basal ganglia, % /mmHg, mean (SD)** | 0.16 (0.049) | 0.17 (0.053) | 0.17 (0.053) |
| **CVR, WMH, % /mmHg, mean (SD)** | 0.043 (0.043) | 0.040 (0.043) | 0.041 (0.043) |
|  |  |  |  |
|  |  |  |  |

^*In participants with blood biomarker data, longitudinal 1-year imaging data were available for 163 participants and cognitive data for 162 participants.^

**Abbreviations:** **IQR:** interquartile range; **NIHSS:** The National Institutes of Health Stroke Scale; **NART**: National Adult Reading Test; **WMH**: white matter hyperintensity; **ICV:** intracranial volume; **SVD**: small vessel disease; **DWI:** diffusion-weighted imaging; **MoCA:** Montreal Cognitive Assessment; **PDGFRβ:** platelet derived growth factor receptor beta; **PDGF-BB:** platelet derived growth factor subunit B; **VCAM-1:** vascular cell adhesion molecule 1; **ICAM-1:** intercellular adhesion molecule 1; **E-selectin:** endothelial-selectin; **P-Selectin:** platelet-selectin; **Endo-1:** Endothelin-1; **TNF-α:** tumour necrosis factor alpha; **vWF:** von Willebrand Factor; **MMP-9:** matrix metalloproteinase 9; **IL-6:** Interleukin 6; **VEGF:** vascular endothelial growth factor; **PLGF:** placental growth factor; **PS:** permeability-surface area product ; **NAWM:** normal-appearing white matter; **V_p_:** blood plasma volume fraction; **CVR:** cerebrovascular reactivity; **TMT:** trail making test.

**Table S2. Baseline cohort characteristics of patients with blood biomarker data and one year follow-up data compared to patients without one year follow-up data.**

|  | | |
| --- | --- | --- |
|  | **No (N=18)** | **Yes (N=163)** |
| **Sex, female, n (%)** | 5 (28 %) | 52 (32 %) |
| **Age, years, mean (SD)** | 65 (± 12) | 65 (± 11) |
| **Stroke subtype, lacunar, n (%)** | 9 (50 %) | 97 (60 %) |
| **Diabetes, yes, n (%)** | 6 (33 %) | 37 (23 %) |
| **Hypertension, yes, n (%)** | 12 (67 %) | 114 (70 %) |
| **Hypercholesterolaemia, yes, n (%)** | 13 (72 %) | 120 (74 %) |
| **Smoking status, current smoker, n (%)** | 5 (28 %) | 18 (11 %) |
| **Risk factor score, mean (SD)** | 3.0 (± 1.3) | 2.8 (± 0.92) |
| **Baseline NIHSS, mean (SD)** | 2.0 (± 0.91) | 2.4 (± 1.5) |
| **Baseline NART score, number of errors, mean (SD)** | 19 (± 9.3) | 17 (± 9.5) |
| **Baseline WMH volume (mm^3^ as % ICV), mean (SD)** | 0.72 (± 0.56) | 0.98 (± 1.2) |
| **1-year follow-up WMH volume (mm^3^ as % ICV), mean (SD)^*^** | NA (± NA) | 1.0 (± 1.3) |
| **Change in WMH volume between baseline and 1-year follow-up (mm^3^ as % ICV), mean (SD)^*^** | NA (± NA) | 0.068 (± 0.20) |
| **Baseline SVD composite score, mean (SD)** | 2.6 (± 1.4) | 2.9 (± 1.3) |
| **1-year follow-up SVD composite score, mean (SD)^*^** | 18 (100%) | 2 (1.2%) |
| **Baseline Fazekas score, mean (SD)** | 3.8 (± 1.4) | 4.1 (± 1.6) |
| **1-year follow-up Fazekas score, mean (SD)^*^** | NA (± NA) | 4.0 (± 1.7) |
| **Lacunes at baseline, number, median (IQR)** | 0 (0,2) | 1 (0,3) |
| **Lacunes at 1-year follow-up, number, median (IQR)^*^** | NA (± NA) | 1 (0,3) |
| **Microbleeds at baseline, number, median (IQR)** | 0 (0,1) | 0 (0,0) |
| **Microbleeds at 1-year follow-up, number, median (IQR)^*^** | NA (± NA) | 0 (0,0) |
| **New DWI-positive lesions between baseline and 1-year follow-up detected, number, mean (SD)^*^** | 0.33 (± 0.97) | 0.56 (± 1.2) |
| **MoCA score at baseline, mean (SD)** | 25 (± 2.5) | 25 (± 3.6) |
| **MoCA score at 1-year follow-up, mean (SD)^*^** | 22 (± 6.0) | 26 (± 3.6) |
| **Baseline Trail Making Test ratio (A/B), mean (SD)** | 2.8 (0.82) | 2.9 (1.3) |
| **1-year follow-up Trail Making Test ratio (A/B), mean (SD)^*^** | 2.3 (1.1) | 2.8 (1.3) |
| **PDGFRβ, mean (SD)** | 57000 (± 24000) | 57000 (± 23000) |
| **PDGF-BB, mean (SD)** | 9900 (± 4000) | 8800 (± 3200) |
| **VCAM-1, mean (SD)** | 2300 (± 720) | 2100 (± 760) |
| **ICAM-1, mean (SD)** | 220000 (± 100000) | 200000 (± 80000) |
| **P-Selectin, mean (SD)** | 130 (± 59) | 110 (± 47) |
| **E-Selectin, mean (SD)** | 52 (± 22) | 42 (± 19) |
| **Endo-1, mean (SD)** | 5.0 (± 4.5) | 3.6 (± 2.5) |
| **TNF-α, mean (SD)** | 1.2 (± 0.67) | 1.1 (± 0.73) |
| **VWF, mean (SD)** | 1400 (± 620) | 1300 (± 820) |
| **MMP-9, mean (SD)** | 870 (± 350) | 870 (± 450) |
| **IL-6, mean (SD)** | 7.0 (± 16) | 3.9 (± 8.5) |
| **VEGF, mean (SD)** | 300 (± 190) | 350 (± 200) |
| **PLGF, mean (SD)** | 9.0 (± 3.2) | 9.6 (± 2.9) |
| **PS, NAWM, x10^4^, mean (SD)** | 0.20 (± 1.1) | 0.15 (± 1.0) |
| **PS, basal ganglia, x10^4^, mean (SD)** | 0.60 (± 1.4) | 0.76 (± 1.3) |
| **PS, WMH, x10^4^, mean (SD)** | 1.1 (± 1.2) | 0.82 (± 1.6) |
| **V_p_, NAWM, x10^3^, mean (SD)** | 5.4 (± 1.6) | 6.3 (± 1.8) |
| **V_p_, basal ganglia, x10^3^, mean (SD)** | 13 (± 1.3) | 15 (± 3.6) |
| **V_p_, WMH, x10^3^, mean (SD)** | 7.3 (± 3.8) | 8.5 (±3.7) |
| **CVR, NAWM, % /mmHg, mean (SD)** | 0.046 (0.016) | 0.043 (0.017) |
| **CVR, basal ganglia, % /mmHg, mean (SD)** | 0.19 (0.050) | 0.17 (0.053) |
| **CVR, WMH, % /mmHg, mean (SD)** | 0.038 (0.038) | 0.040 (0.044) |
|  |  |  |

^*In participants with blood biomarker data, longitudinal 1-year imaging data were available for 163 participants and cognitive data for 162 participants.^

**Abbreviations:** **IQR:** interquartile range; **NIHSS:** The National Institutes of Health Stroke Scale; **NART**: National Adult Reading Test; **WMH**: white matter hyperintensity; **ICV:** intracranial volume; **SVD**: small vessel disease; **DWI:** diffusion-weighted imaging; **MoCA:** Montreal Cognitive Assessment; **PDGFRβ:** platelet derived growth factor receptor beta; **PDGF-BB:** platelet derived growth factor subunit B; **VCAM-1:** vascular cell adhesion molecule 1; **ICAM-1:** intercellular adhesion molecule 1; **E-selectin:** endothelial-selectin; **P-Selectin:** platelet-selectin; **Endo-1:** Endothelin-1; **TNF-α:** tumour necrosis factor alpha; **vWF:** von Willebrand Factor; **MMP-9:** matrix metalloproteinase 9; **IL-6:** Interleukin 6; **VEGF:** vascular endothelial growth factor; **PLGF:** placental growth factor; **PS:** permeability-surface area product ; **NAWM:** normal-appearing white matter; **V_p_:** blood plasma volume fraction; **CVR:** cerebrovascular reactivity; **TMT:** trail making test.

**Table S3. Baseline cohort characteristics of lacunar stroke patients compared to cortical stroke patients with blood biomarker data.**

|  | | |
| --- | --- | --- |
|  | **Lacunar (N=106)** | **Cortical (N=74)** |
| **Sex, female, n (%)** | 31 (29 %) | 25 (34 %) |
| **Age, years, mean (SD)** | 64 (± 11) | 67 (± 11) |
| **Diabetes, yes, n (%)** | 30 (28 %) | 13 (18 %) |
| **Hypertension, yes, n (%)** | 78 (74 %) | 48 (65 %) |
| **Hypercholesterolaemia, yes, n (%)** | 84 (79 %) | 49 (66 %) |
| **Smoking status, current smoker, n (%)** | 16 (15 %) | 7 (9 %) |
| **Risk factor score, mean (SD)** | 3.0 (± 0.90) | 2.6 (± 0.98) |
| **Baseline NIHSS, mean (SD)** | 2.6 (± 1.5) | 2.1 (± 1.2) |
| **Baseline NART score, number of errors, mean (SD)** | 17 (± 9.3) | 18 (± 9.8) |
| **Baseline WMH volume (mm^3^ as % ICV), mean (SD)** | 1.0 (± 1.2) | 0.84 (± 1.1) |
| **1-year follow-up WMH (mm3 as % ICV), mean (SD)** | 1.1 (± 1.3) | 0.91 (± 1.2) |
| **Change in WMH volume between baseline and 1-year follow-up (mm3 as % ICV), mean (SD)** | 0.060 (± 0.18) | 0.079 (± 0.22) |
| **Baseline SVD composite score, mean (SD)** | 3.1 (± 1.3) | 2.5 (± 1.3) |
| **1-year follow-up SVD composite score, mean (SD)** | 3.0 (± 1.3) | 2.5 (± 1.2) |
| **Baseline Fazekas score, mean (SD)** | 4.2 (± 1.6) | 3.9 (± 1.6) |
| **1-year follow-up Fazekas score, mean (SD)** | 4.2 (± 1.7) | 3.8 (± 1.7) |
| **Lacunes at baseline, number, median (IQR)** | 2.8 (± 3.8) | 0.95 (± 1.7) |
| **Lacunes at 1-year follow-up, number, median (IQR)** | 3.0 (± 3.9) | 0.98 (± 1.8) |
| **Microbleeds at baseline, number, median (IQR)** | 1.9 (± 6.6) | 0.58 (± 2.7) |
| **Microbleeds at 1-year follow-up, number, median (IQR)** | 2.8 (± 8.3) | 0.51 (± 2.6) |
| **New DWI+ lesions between baseline and 1-year follow-up detected, number, mean (SD)** | 0.73 (± 1.4) | 0.28 (± 0.65) |
| **MoCA score at baseline, mean (SD)** | 25 (± 3.4) | 25 (± 3.7) |
| **MoCA score at 1-year follow-up, mean (SD)** | 26 (± 3.5) | 25 (± 3.7) |
| **Baseline Trail Making Test ratio (A/B), mean (SD)** | 2.8 (± 1.3) | 3.0 (± 1.2) |
| **PDGFRβ, mean (SD)** | 55000 (± 24000) | 59000 (± 22000) |
| **PDGF_BB, mean (SD)** | 9100 (± 3200) | 8500 (± 3300) |
| **VCAM-1, mean (SD)** | 2100 (± 810) | 2200 (± 690) |
| **ICAM-1, mean (SD)** | 200000 (± 79000) | 200000 (± 87000) |
| **P-Selectin, mean (SD)** | 110 (± 39) | 120 (± 60) |
| **E-Selectin, mean (SD)** | 45 (± 22) | 41 (± 16) |
| **Endo-1, mean (SD)** | 3.9 (± 3.0) | 3.6 (± 2.5) |
| **TNF-α, mean (SD)** | 1.2 (± 0.81) | 1.1 (± 0.59) |
| **VWF, mean (SD)** | 1300 (± 860) | 1300 (± 720) |
| **MMP-9, mean (SD)** | 910 (± 400) | 810 (± 480) |
| **IL-6, mean (SD)** | 4.9 (± 10) | 3.2 (± 8.0) |
| **VEGF, mean (SD)** | 370 (± 210) | 310 (± 190) |
| **PLGF, mean (SD)** | 9.7 (± 3.0) | 9.4 (± 2.7) |
| **PS, NAWM, x10^4^, mean (SD)** | 0.097 (± 1.0) | 0.25 (± 1.0) |
| **PS, basal ganglia, x10^4^, mean (SD)** | 0.81 (± 1.3) | 0.67 (± 1.2) |
| **PS, WMH, x10^4^, mean (SD)** | 0.57 (± 1.5) | 1.2 (± 1.6) |
| **V_p_, NAWM, x10^3^, mean (SD)** | 6.1 (± 1.8) | 6.4 (± 1.9) |
| **V_p_, basal ganglia, x10^3^, mean (SD)** | 14 (± 3.5) | 15 (± 3.4) |
| **V_p_, WMH, x10^3^, mean (SD)** | 7.9 (± 3.4) | 9.2 (± 4.0) |
| **CVR, NAWM, % /mmHg, mean (SD)** | 0.042 (± 0.017) | 0.045 (± 0.016) |
| **CVR, basal ganglia, % /mmHg, mean (SD)** | 0.17 (± 0.054) | 0.18 (± 0.052) |

**Abbreviations:** **IQR:** interquartile range; **NIHSS:** The National Institutes of Health Stroke Scale; **NART**: National Adult Reading Test; **WMH**: white matter hyperintensity; **ICV:** intracranial volume; **SVD**: small vessel disease; **DWI:** diffusion-weighted imaging; **MoCA:** Montreal Cognitive Assessment; **PDGFRβ:** platelet derived growth factor receptor beta; **PDGF-BB:** platelet derived growth factor subunit B; **VCAM-1:** vascular cell adhesion molecule 1; **ICAM-1:** intercellular adhesion molecule 1; **E-selectin:** endothelial-selectin; **P-Selectin:** platelet-selectin; **Endo-1:** Endothelin-1; **TNF-α:** tumour necrosis factor alpha; **vWF:** von Willebrand Factor; **MMP-9:** matrix metalloproteinase 9; **IL-6:** Interleukin 6; **VEGF:** vascular endothelial growth factor; **PLGF:** placental growth factor; **PS:** permeability-surface area product ; **NAWM:** normal-appearing white matter; **V_p_:** blood plasma volume fraction; **CVR:** cerebrovascular reactivity; **TMT:** trail making test.

**Table S4. Pearson correlation between individual biomarkers and the time between stroke onset and serology.**

|  | **Pearson’s coefficient** | **p-value** | **95% CI** |
| --- | --- | --- | --- |
| **PDGFRβ** | -0.052 | 0.489 | -0.197, 0.953 |
| **PDGF-BB** | -0.051 | 0.493 | -0.196, 0.095 |
| **VCAM-1** | 0.121 | 0.105 | -0.025, 0.263 |
| **ICAM-1** | -0.001 | 0.989 | -0.147, 0.145 |
| **P-Selectin** | 0.078 | 0.296 | -0.068, 0.222 |
| **E-Selectin** | -0.139 | 0.067 | -0.283, 0.010 |
| **Endo-1** | 0.104 | 0.173 | -0.046, 0.250 |
| **TNF-α** | -0.023 | 0.755 | -0.169, 0.123 |
| **vWF** | 0.021 | 0.773 | -0.125, 0.167 |
| **MMP-9** | -0.044 | 0.559 | -0.193, 0.105 |
| **IL-6** | 0.079 | 0.291 | -0.068, 0.223 |
| **VEGF** | -0.019 | 0.795 | -0.165, 0.127 |
| **PLGF** | 0.102 | 0.171 | -0.044, 0.245 |

**Abbreviations: CI:** confidence interval; **PDGFRβ:** platelet derived growth factor receptor beta; **PDGF-BB:** platelet derived growth factor subunit B; **VCAM-1:** vascular cell adhesion molecule 1; **ICAM-1:** intercellular adhesion molecule 1; **E-selectin:** endothelial-selectin; **P-Selectin:** platelet-selectin; **Endo-1:** Endothelin-1; **TNF-α:** tumour necrosis factor alpha; **vWF:** von Willebrand Factor; **MMP-9:** matrix metalloproteinase 9; **IL-6:** Interleukin 6; **VEGF:** vascular endothelial growth factor; **PLGF:** placental growth factor.

**Table S5. Blood biomarkers and WMH volume.** Linear regression results for the association between distinct standardised blood biomarkers and log transformed WMH volume as % ICV at baseline (linear regression) and one year follow-up (linear mixed effects model) after adjusting for age, sex, vascular risk factors, stroke subtype, and baseline NIHSS.

|  | *Baseline* | | *Longitudinal WMH volume* | |
| --- | --- | --- | --- | --- |
|  | **β** | **95% CI** | **β** | **95% CI** |
| **PDGFRβ** | -0.025 | -0.174, 0.123 | -0.070 | -0.216, 0.076 |
| **PDGF-BB** | 0.108 | -0.041, 0.257 | 0.114 | -0.035, 0.263 |
| **VCAM-1** | -0.133 | -0.292, 0.024 | -0.168 | -0.326, 0.010 |
| **ICAM-1** | -0.026 | -0.175, 0.122 | -0.025 | -0.174, 0.123 |
| **P-Selectin** | -0.115 | -0.258, 0.028 | -0.116 | -0.260, 0.028 |
| **E-Selectin** | 0.028 | -0.123, 0.179 | 0.020 | -0.131, 0.173 |
| **Endo-1** | 0.068 | -0.077, 0.215 | 0.062 | -0.085, 0.210 |
| **TNF-α** | 0.001 | -0.151, 0.152 | 0.007 | -0.143, 0.159 |
| **vWF** | -0.115 | -0.266, 0.035 | -0.138 | -0.288, 0.011 |
| **MMP-9** | 0.090 | -0.057, 0.238 | 0.107 | -0.040, 0.256 |
| **IL-6** | -0.007 | -0.152, 0.137 | 0.000 | -0.144, 0.145 |
| **VEGF** | -0.025 | -0.172, 0.122 | -0.026 | -0.174, 0.120 |
| **PLGF** | -0.008 | -0.153, 0.137 | 0.005 | -0.139, 0.151 |

**Abbreviations: WMH:** white matter hyperintensity; **ICV:** intracranial volume; **NIHSS:** The National Institutes of Health Stroke Scale; **CI:** confidence interval; **PDGFRβ:** platelet derived growth factor receptor beta; **PDGF-BB:** platelet derived growth factor subunit B; **VCAM-1:** vascular cell adhesion molecule 1; **ICAM-1:** intercellular adhesion molecule 1; **E-selectin:** endothelial-selectin; **P-Selectin:** platelet-selectin; **Endo-1:** Endothelin-1; **TNF-α:** tumour necrosis factor alpha; **vWF:** von Willebrand Factor; **MMP-9:** matrix metalloproteinase 9; **IL-6:** Interleukin 6; **VEGF:** vascular endothelial growth factor; **PLGF:** placental growth factor.

**Table S6. Blood biomarkers and Fazekas score.** Ordinal regression results for the association of distinct standardised blood biomarkers and Fazekas score at baseline after adjusting for age, sex, vascular risk factors, stroke subtype, and baseline NIHSS.

|  | *Baseline* | |
| --- | --- | --- |
|  | **OR** | **95% CI** |
| **PDGFRβ** | 0.959 | 0.727, 1.263 |
| **PDGF-BB** | 1.155 | 0.862, 1.547 |
| **VCAM-1** | 0.877 | 0.648, 1.187 |
| **ICAM-1** | 1.090 | 0.815, 1.455 |
| **P-Selectin** | 0.826 | 0.624, 1.096 |
| **E-Selectin** | 1.121 | 0.841, 1.511 |
| **Endo-1** | 0.906 | 0.691, 1.195 |
| **TNF-α** | 0.872 | 0.641, 1.150 |
| **vWF** | 0.824 | 0.607, 1.137 |
| **MMP-9** | 1.045 | 0.786, 1.410 |
| **IL-6** | 0.974 | 0.757, 1.249 |
| **VEGF** | 0.991 | 0.751, 1.310 |
| **PLGF** | 1.000 | 0.752, 1.329 |

**Abbreviations:** **NIHSS:** The National Institutes of Health Stroke Scale; **CI:** confidence interval; **PDGFRβ:** platelet derived growth factor receptor beta; **PDGF-BB:** platelet derived growth factor subunit B; **VCAM-1:** vascular cell adhesion molecule 1; **ICAM-1:** intercellular adhesion molecule 1; **E-selectin:** endothelial-selectin; **P-Selectin:** platelet-selectin; **Endo-1:** Endothelin-1; **TNF-α:** tumour necrosis factor alpha; **vWF:** von Willebrand Factor; **MMP-9:** matrix metalloproteinase 9; **IL-6:** Interleukin 6; **VEGF:** vascular endothelial growth factor; **PLGF:** placental growth factor.

**Table S7. Blood biomarkers and the presence of lacunes and/or microbleeds at baseline.** Logistic regression results for the association of distinct standardised blood biomarkers and the prevalence of lacunes and microbleeds at baseline after adjusting for age, sex, vascular risk factors, and stroke subtype.

|  | *Baseline* | | | |
| --- | --- | --- | --- | --- |
|  | ***Lacunes*** | | ***Microbleeds*** | |
|  | **OR** | **95% CI (exp)** | **OR** | **95% CI (exp)** |
| **PDGFRβ** | 0.715 | 0.508, 0.993 | 0.823 | 0.548, 1.222 |
| **PDGF-BB** | 0.921 | 0.654, 1.285 | 1.058 | 0.690, 1.607 |
| **VCAM-1** | 0.920 | 0.652, 1.307 | 0.910 | 0.587, 1.367 |
| **ICAM-1** | 1.259 | 0.909, 1.770 | 0.860 | 0.557, 1.282 |
| **P-Selectin** | 0.967 | 0.697, 1.328 | 0.841 | 0.519, 1.270 |
| **E-Selectin** | 1.153 | 0.817, 1.665 | 0.656 | 0.386, 1.023 |
| **Endo-1** | 0.713 | 0.496, 0.990 | 1.004 | 0.440, 1.989 |
| **TNF-α** | 0.842 | 0.516, 1.314 | 0.794 | 0.483, 1.178 |
| **vWF** | 1.140 | 0.812, 1.639 | 0.699 | 0.427, 1.057 |
| **MMP-9** | 0.814 | 0.565, 1.146 | 1.061 | 0.672, 1.597 |
| **IL-6** | 0.817 | 0.542, 1.118 | 0.739 | 0.251, 1.176 |
| **VEGF** | 0.914 | 0.662, 1.258 | 1.267 | 0.864, 1.856 |
| **PLGF** | 0.928 | 0.676, 1.272 | 0.887 | 0.591, 1.291 |

**Abbreviations:** **CI:** confidence interval; **PDGFRβ:** platelet derived growth factor receptor beta; **PDGF-BB:** platelet derived growth factor subunit B; **VCAM-1:** vascular cell adhesion molecule 1; **ICAM-1:** intercellular adhesion molecule 1; **E-selectin:** endothelial-selectin; **P-Selectin:** platelet-selectin; **Endo-1:** Endothelin-1; **TNF-α:** tumour necrosis factor alpha; **vWF:** von Willebrand Factor; **MMP-9:** matrix metalloproteinase 9; **IL-6:** Interleukin 6; **VEGF:** vascular endothelial growth factor; **PLGF:** placental growth factor.

**Table S8. Blood biomarkers and newly appearing incident (DWI-positive) lesions.** Logistic regression results for the association of distinct standardised blood biomarkers to the prevalence of DWI-positive incident lesions identified throughout the one-year follow-up period after adjusting for age, sex, vascular risk factors, stroke subtype, and baseline NIHSS.

|  | ***New DWI-positive lesion*** | |
| --- | --- | --- |
|  | **OR** | **95% CI** |
| **PDGFRβ** | 0.763 | 0.472, 1.211 |
| **PDGF-BB** | 1.398 | 0.846, 2.328 |
| **VCAM-1** | 0.971 | 0.569, 1.605 |
| **ICAM-1** | 1.274 | 0.840, 1.927 |
| **P-Selectin** | 1.498 | 0.996, 2.351 |
| **E-Selectin** | 0.734 | 0.431, 1.142 |
| **Endo-1** | 0.968 | 0.539, 1.603 |
| **TNF-α** | 0.928 | 0.519, 1.446 |
| **vWF** | 1.063 | 0.672, 1.602 |
| **MMP-9** | 1.148 | 0.678, 1.842 |
| **IL-6** | 0.881 | 0.333, 1.359 |
| **VEGF** | 1.643 | 1.040, 2.624 |
| **PLGF** | 1.335 | 0.826, 2.124 |

**Abbreviations:** **DWI:** diffusion-weighted imaging; **NIHSS:** The National Institutes of Health Stroke Scale; **CI:** confidence interval; **PDGFRβ:** platelet derived growth factor receptor beta; **PDGF-BB:** platelet derived growth factor subunit B; **VCAM-1:** vascular cell adhesion molecule 1; **ICAM-1:** intercellular adhesion molecule 1; **E-selectin:** endothelial-selectin; **P-Selectin:** platelet-selectin; **Endo-1:** Endothelin-1; **TNF-α:** tumour necrosis factor alpha; **vWF:** von Willebrand Factor; **MMP-9:** matrix metalloproteinase 9; **IL-6:** Interleukin 6; **VEGF:** vascular endothelial growth factor; **PLGF:** placental growth factor.

**Table S9. Blood biomarkers and summary SVD score.** Ordinal regression results for the association of distinct standardised blood biomarkers to baseline summary SVD scores after adjusting for age, sex, vascular risk factors, and stroke subtype.

|  | *Baseline* | |
| --- | --- | --- |
|  | **OR** | **95% CI** |
| **PDGFRβ** | 0.845 | 0.639, 1.116 |
| **PDGF-BB** | 1.173 | 0.876, 1.568 |
| **VCAM-1** | 0.933 | 0.696, 1.251 |
| **ICAM-1** | 1.179 | 0.886, 1.571 |
| **P-Selectin** | 0.964 | 0.739, 1.251 |
| **E-Selectin** | 1.111 | 0.857, 1.473 |
| **Endo-1** | 0.840 | 0.639, 1.106 |
| **TNF-α** | 0.779 | 0.580, 1.027 |
| **vWF** | 0.985 | 0.731, 1.327 |
| **MMP-9** | 0.975 | 0.717, 1.317 |
| **IL-6** | 0.883 | 0.688, 1.134 |
| **VEGF** | 1.002 | 0.747, 1.348 |
| **PLGF** | 0.875 | 0.657, 1.160 |

**Abbreviations:** **SVD:** small vessel disease; **CI:** confidence interval; **PDGFRβ:** platelet derived growth factor receptor beta; **PDGF-BB:** platelet derived growth factor subunit B; **VCAM-1:** vascular cell adhesion molecule 1; **ICAM-1:** intercellular adhesion molecule 1; **E-selectin:** endothelial-selectin; **P-Selectin:** platelet-selectin; **Endo-1:** Endothelin-1; **TNF-α:** tumour necrosis factor alpha; **vWF:** von Willebrand Factor; **MMP-9:** matrix metalloproteinase 9; **IL-6:** Interleukin 6; **VEGF:** vascular endothelial growth factor; **PLGF:** placental growth factor.

**Table S10. Blood biomarkers and BBB permeability (DCE-MRI, PS).** Linear regression results for the association of distinct standardised blood biomarkers to baseline DCE-MRI parameter PS (x10^4^) after adjusting for age, sex, vascular risk factors, and baseline WMH volume as % ICV.

| PS | **NAWM** | | **Basal Ganglia** | | **WMH (ventricles removed)** | |
| --- | --- | --- | --- | --- | --- | --- |
|  | **β** | **95% CI** | **β** | **95% CI** | **β** | **95% CI** |
| **PDGFRβ** | -0.001 | -0.171, 0.169 | 0.020 | -0.193, 0.234 | -0.145 | -0.417, 0.126 |
| **PDGF-BB** | -0.124 | -0.290, 0.041 | -0.062 | -0.272, 0.147 | -0.115 | -0.383, 0.152 |
| **VCAM-1** | 0.181 | -0.018, 0.381 | 0.290 | 0.040, 0.539 | 0.032 | -0.290, 0.355 |
| **ICAM-1** | 0.128 | -0.058, 0.316 | 0.039 | -0.197, 0.276 | 0.128 | -0.173, 0.429 |
| **P-Selectin** | 0.043 | -0.114, 0.200 | 0.070 | -0.127, 0.268 | -0.001 | -0.254, 0.251 |
| **E-Selectin** | -0.035 | -0.207, 0.135 | 0.034 | -0.180, 0.248 | -0.160 | -0.434, 0.112 |
| **Endo-1** | 0.051 | -0.131, 0.234 | 0.187 | -0.041, 0.415 | -0.002 | -0.294, 0.288 |
| **TNF-α** | 0.020 | -0.158, 0.199 | 0.248 | 0.027, 0.468 | -0.003 | -0.289, 0.283 |
| **vWF** | 0.123 | -0.080, 0.328 | 0.256 | 0.002, 0.511 | 0.138 | -0.189, 0.466 |
| **MMP-9** | -0.070 | -0.234, 0.094 | 0.033 | -0.172, 0.240 | -0.080 | -0.344, 0.183 |
| **IL-6** | 0.066 | -0.103, 0.235 | 0.115 | -0.096, 0.328 | -0.063 | -0.335, 0.208 |
| **VEGF** | 0.015 | -0.146, 0.177 | 0.058 | -0.143, 0.261 | 0.006 | -0.252, 0.266 |
| **PLGF** | 0.190 | 0.027, 0.354 | 0.159 | -0.047, 0.367 | 0.109 | -0.156, 0.375 |

**Abbreviations: DCE-MRI:** dynamic contrast-enhanced magnetic resonance imaging; **PS:** Permeability-Surface area product; **WMH:** white matter hyperintensity; **ICV:** intracranial volume; **CI:** confidence interval; **PDGFRβ:** platelet derived growth factor receptor beta; **PDGF-BB:** platelet derived growth factor subunit B; **VCAM-1:** vascular cell adhesion molecule 1; **ICAM-1:** intercellular adhesion molecule 1; **E-selectin:** endothelial-selectin; **P-Selectin:** platelet-selectin; **Endo-1:** Endothelin-1; **TNF-α:** tumour necrosis factor alpha; **vWF:** von Willebrand Factor; **MMP-9:** matrix metalloproteinase 9; **IL-6:** Interleukin 6; **VEGF:** vascular endothelial growth factor; **PLGF:** placental growth factor.

**Table S11. Blood biomarkers and BBB permeability (DCE-MRI, V_p_).** Linear regression results for the association of distinct standardised blood biomarkers to baseline DCE-MRI parameter V_p_ (x10^3^) after adjusting for age, sex, vascular risk factors, and baseline WMH volume as % ICV.

| V_P_ | **NAWM** | | **Basal Ganglia** | | **WMH (ventricles removed)** | |
| --- | --- | --- | --- | --- | --- | --- |
|  | **β** | **95% CI** | **β** | **95% CI** | **β** | **95% CI** |
| **PDGFRβ** | -0.047 | -0.351, 0.257 | -0.159 | -0.741, 0.423 | -0.152 | -0.717, 0.411 |
| **PDGF-BB** | 0.081 | -0.217, 0.379 | 0.457 | -0.110, 1.025 | -0.087 | -0.643, 0.467 |
| **VCAM-1** | 0.031 | -0.328, 0.392 | 0.070 | -0.619, 0.760 | -0.283 | -0.951, 0.384 |
| **ICAM-1** | 0.126 | -0.209, 0.463 | 0.417 | -0.224, 1.059 | -0.036 | -0.662, 0.588 |
| **P-Selectin** | -0.037 | -0.319, 0.244 | 0.003 | -0.537, 0.543 | -0.031 | -0.554, 0.492 |
| **E-Selectin** | -0.060 | -0.365, 0.244 | -0.187 | -0.768, 0.394 | -0.501 | -1.060, 0.058 |
| **Endo-1** | -0.174 | -0.499, 0.150 | -0.337 | -0.954, 0.279 | -0.102 | -0.700, 0.495 |
| **TNF-α** | -0.063 | -0.382, 0.255 | -0.268 | -0.878, 0.341 | 0.199 | -0.392, 0.791 |
| **vWF** | 0.113 | -0.253, 0.479 | 0.128 | -0.573, 0.830 | 0.128 | -0.573, 0.830 |
| **MMP-9** | 0.026 | -0.267, 0.319 | -0.140 | -0.700, 0.419 | -0.049 | -0.593, 0.494 |
| **IL-6** | -0.372 | -0.669, 0.074 | -0.543 | -1.118, 0.030 | -0.242 | -0.804, 0.320 |
| **VEGF** | 0.033 | -0.255, 0.322 | 0.091 | -0.461, 0.644 | -0.197 | -0.732, 0.338 |
| **PLGF** | -0.249 | -0.544, 0.044 | -0.322 | -0.889, 0.244 | -0.338 | -0.887, 0.211 |

**Abbreviations: DCE-MRI:** dynamic contrast-enhanced magnetic resonance imaging; **V_p_:** blood plasma volume fraction; **WMH:** white matter hyperintensity; **ICV:** intracranial volume; **CI:** confidence interval; **PDGFRβ:** platelet derived growth factor receptor beta; **PDGF-BB:** platelet derived growth factor subunit B; **VCAM-1:** vascular cell adhesion molecule 1; **ICAM-1:** intercellular adhesion molecule 1; **E-selectin:** endothelial-selectin; **P-Selectin:** platelet-selectin; **Endo-1:** Endothelin-1; **TNF-α:** tumour necrosis factor alpha; **vWF:** von Willebrand Factor; **MMP-9:** matrix metalloproteinase 9; **IL-6:** Interleukin 6; **VEGF:** vascular endothelial growth factor; **PLGF:** placental growth factor.

**Table S12. Blood biomarkers and CVR.** Linear regression results for the association of distinct standardised blood biomarkers to baseline DCE-CVR in NAWM, the basal ganglia and WMH after adjusting for age, sex, vascular risk factors, and baseline WMH volume as % ICV.

| CVR | **NAWM** | | **Basal Ganglia** | | **WMH (ventricles removed)** | |
| --- | --- | --- | --- | --- | --- | --- |
|  | **β** | **95% CI** | **β** | **95% CI** | **β** | **95% CI** |
| **PDGFRβ** | 0.002 | -0.001, 0.004 | 0.005 | -0.003, 0.014 | -0.001 | -0.008, 0.007 |
| **PDGF-BB** | 0.002 | -0.000, 0.005 | 0.012 | 0.003, 0.021 | 0.002 | -0.005, 0.010 |
| **VCAM-1** | -0.001 | -0.004, 0.001 | -0.005 | -0.014, 0.004 | -0.008 | -0.016, -0.000 |
| **ICAM-1** | -0.002 | -0.004, 0.001 | -0.010 | -0.019, -0.001 | -0.002 | -0.010, 0.004 |
| **P-Selectin** | 0.002 | -0.001, 0.005 | 0.011 | -0.001, 0.022 | 0.005 | -0.003, 0.014 |
| **E-Selectin** | 0.001 | -0.003, 0.002 | -0.002 | -0.012, 0.007 | -0.001 | -0.008, 0.008 |
| **Endo-1** | -0.001 | -0.003, 0.002 | 0.009 | 0.001, 0.018 | -0.005 | -0.012, 0.002 |
| **TNF-α** | -0.001 | -0.005, 0.001 | 0.007 | -0.003, 0.018 | -0.001 | -0.010, 0.008 |
| **vWF** | -0.001 | -0.003, 0.001 | 0.000 | -0.008, 0.008 | -0.004 | -0.011, 0.002 |
| **MMP-9** | 0.001 | -0.001, 0.004 | 0.008 | -0.001, 0.017 | 0.004 | -0.003, 0.012 |
| **IL-6** | -0.001 | -0.003, 0.001 | -0.001 | -0.007, 0.007 | 0.000 | -0.007, 0.007 |
| **VEGF** | -0.000 | -0.002, 0.002 | 0.001 | -0.007, 0.009 | -0.005 | -0.012, 0.001 |
| **PLGF** | -0.001 | -0.004, 0.001 | 0.000 | -0.008, 0.008 | 0.004 | -0.002, 0.012 |

**Abbreviations: MRI:** magnetic resonance imaging; **CVR:** cerebrovascular reactivity; **NAWM:** normal appearing white matter; **WMH:** white matter hyperintensity; **ICV:** intracranial volume; **CI:** confidence interval; **PDGFRβ:** platelet derived growth factor receptor beta; **PDGF-BB:** platelet derived growth factor subunit B; **VCAM-1:** vascular cell adhesion molecule 1; **ICAM-1:** intercellular adhesion molecule 1; **E-selectin:** endothelial-selectin; **P-Selectin:** platelet-selectin; **Endo-1:** Endothelin-1; **TNF-α:** tumour necrosis factor alpha; **vWF:** von Willebrand Factor; **MMP-9:** matrix metalloproteinase 9; **IL-6:** Interleukin 6; **VEGF:** vascular endothelial growth factor; **PLGF:** placental growth factor.

**Table S13. Blood biomarkers and MoCA score.** Baseline cross-sectional linear regression and longitudinal linear mixed effects model results for the association of distinct standardised blood biomarkers and MoCA score after adjusting for age, sex, vascular risk factors, premorbid intelligence, and baseline WMH volume as % ICV.

|  | *Baseline* | | *One year follow-up* | |
| --- | --- | --- | --- | --- |
|  | **β** | **95% CI** | **β** | **95% CI** |
| **PDGFRβ** | 0.152 | -0.315, 0.620 | 0.005 | -0.396, 0.407 |
| **PDGF-BB** | -0.087 | -0.568, 0.393 | -0.083 | -0.493, 0.325 |
| **VCAM-1** | -0.328 | -0.845, 0.188 | -0.320 | -0.764, 0.124 |
| **ICAM-1** | 0.149 | -0.333, 0.633 | -0.022 | -0.438, 0.392 |
| **P-Selectin** | 0.229 | -0.230, 0.689 | 0.069 | -0.320, 0.460 |
| **E-Selectin** | 0.336 | -0.156, 0.829 | 0.300 | -0.120, 0.722 |
| **Endo-1** | -0.204 | -0.661, 0.252 | -0.178 | -0.564, 0.207 |
| **TNF-α** | 0.199 | -0.274, 0.673 | 0.163 | -0.241, 0.568 |
| **vWF** | 0.157 | -0.334, 0.650 | 0.041 | -0.380, 0.463 |
| **MMP-9** | -0.112 | -0.596, 0.370 | 0.108 | -0.303, 0.521 |
| **IL-6** | 0.047 | -0.402, 0.496 | 0.154 | -0.241, 0.550 |
| **VEGF** | -0.346 | -0.798, 0.106 | -0.161 | -0.550, 0.228 |
| **PLGF** | -0.213 | -0.673, 0.247 | -0.146 | -0.539, 0.247 |

**Abbreviations:** **MoCA:** Montreal Cognitive Assessment; **WMH:** white matter hyperintensity; **ICV:** intracranial volume; **CI:** confidence interval; **PDGFRβ:** platelet derived growth factor receptor beta; **PDGF-BB:** platelet derived growth factor subunit B; **VCAM-1:** vascular cell adhesion molecule 1; **ICAM-1:** intercellular adhesion molecule 1; **E-selectin:** endothelial-selectin; **P-Selectin:** platelet-selectin; **Endo-1:** Endothelin-1; **TNF-α:** tumour necrosis factor alpha; **vWF:** von Willebrand Factor; **MMP-9:** matrix metalloproteinase 9; **IL-6:** Interleukin 6; **VEGF:** vascular endothelial growth factor; **PLGF:** placental growth factor.

**Table S14. Blood biomarkers and MCI.** Logistic regression results for the association of distinct standardised blood biomarkers and MCI prevalence at baseline and one year follow-up after adjusting for age, sex, vascular risk factors, premorbid intelligence, and baseline WMH volume as % ICV. One-year cross-sectional models were also adjusted for baseline MCI status.

|  | *Baseline* | | *One year follow-up* | |
| --- | --- | --- | --- | --- |
|  | **OR** | **95% CI** | **OR** | **95% CI** |
| **PDGFRβ** | 0.949 | 0.674, 1.334 | 0.955 | 0.653, 1.390 |
| **PDGF-BB** | 0.982 | 0.693, 1.389 | 1.294 | 0.895, 1.895 |
| **VCAM-1** | 1.480 | 0.962, 2.370 | 1.503 | 0.980, 2.402 |
| **ICAM-1** | 1.052 | 0.727, 1.549 | 1.336 | 0.897, 2.041 |
| **P-Selectin** | 1.206 | 0.816, 1.861 | 1.645 | 1.047, 2.721 |
| **E-Selectin** | 1.109 | 0.748, 1.692 | 1.010 | 0.682, 1.526 |
| **Endo-1** | 1.048 | 0.750, 1.490 | 1.061 | 0.700, 1.609 |
| **TNF-α** | 0.857 | 0.589, 1.212 | 0.884 | 0.576, 1.291 |
| **vWF** | 1.370 | 0.894, 2.260 | 1.247 | 0.844, 1.938 |
| **MMP-9** | 1.458 | 0.977, 2.278 | 0.802 | 0.540, 1.177 |
| **IL-6** | 0.800 | 0.542, 1.094 | 0.385 | 0.073, 0.942 |
| **VEGF** | 1.314 | 0.930, 1.893 | 0.924 | 0.644, 1.323 |
| **PLGF** | 1.136 | 0.804, 1.631 | 0.971 | 0.668, 1.415 |

**Abbreviations:** **MCI:** mild cognitive impairment; **WMH:** white matter hyperintensity; **ICV:** intracranial volume; **CI:** confidence interval; **PDGFRβ:** platelet derived growth factor receptor beta; **PDGF-BB:** platelet derived growth factor subunit B; **VCAM-1:** vascular cell adhesion molecule 1; **ICAM-1:** intercellular adhesion molecule 1; **E-selectin:** endothelial-selectin; **P-Selectin:** platelet-selectin; **Endo-1:** Endothelin-1; **TNF-α:** tumour necrosis factor alpha; **vWF:** von Willebrand Factor; **MMP-9:** matrix metalloproteinase 9; **IL-6:** Interleukin 6; **VEGF:** vascular endothelial growth factor; **PLGF:** placental growth factor.

**Table S15. Blood biomarkers and executive function (TMT ratio B/A).** Baseline cross-sectional linear regression and longitudinal linear mixed effects model results for the association of distinct standardised blood biomarkers and TMT ratio (B/A) after adjusting for age, sex, vascular risk factors, premorbid intelligence, and baseline WMH volume as % ICV.

|  | *Baseline* | | *One year follow-up* | |
| --- | --- | --- | --- | --- |
|  | **β** | **95% CI** | **β** | **95% CI** |
| **PDGFRβ** | -0.047 | -0.225, 0.129 | 0.001 | -0.005, 0.007 |
| **PDGF-BB** | -0.151 | -0.330, 0.028 | -0.030 | -0.077, 0.016 |
| **VCAM-1** | 0.073 | -0.128, 0.274 | 0.005 | -0.215, 0.227 |
| **ICAM-1** | -0.016 | -0.197, 0.165 | 0.001 | -0.001, 0.002 |
| **P-Selectin** | -0.095 | -0.269, 0.078 | -0.016 | -0.164, 0.131 |
| **E-Selectin** | -0.085 | -0.281, 0.110 | 0.042 | -0.121, 0.207 |
| **Endo-1** | 0.110 | -0.062, 0.284 | 0.141 | -0.011, 0.295 |
| **TNF-α** | 0.137 | -0.041, 0.317 | 0.080 | -0.072, 0.233 |
| **vWF** | 0.134 | -0.052, 0.320 | 0.189 | 0.031, 0.347 |
| **MMP-9** | 0.058 | -0.118, 0.234 | 0.008 | -0.147, 0.165 |
| **IL-6** | 0.105 | -0.062, 0.273 | 0.052 | -0.096, 0.201 |
| **VEGF** | -0.163 | -0.334, 0.006 | -0.153 | -0.300, 0.007 |
| **PLGF** | -0.187 | -0.360, -0.015 | -0.096 | -0.245, 0.053 |

**Abbreviations:** **TMT:** Trail Making Task; **WMH:** white matter hyperintensity; **ICV:** intracranial volume; **CI:** confidence interval; **PDGFRβ:** platelet derived growth factor receptor beta; **PDGF-BB:** platelet derived growth factor subunit B; **VCAM-1:** vascular cell adhesion molecule 1; **ICAM-1:** intercellular adhesion molecule 1; **E-selectin:** endothelial-selectin; **P-Selectin:** platelet-selectin; **Endo-1:** Endothelin-1; **TNF-α:** tumour necrosis factor alpha; **vWF:** von Willebrand Factor; **MMP-9:** matrix metalloproteinase 9; **IL-6:** Interleukin 6; **VEGF:** vascular endothelial growth factor; **PLGF:** placental growth factor; **EF:** executive function.

# Supplemental Figures

**Figure S1. Participant flow chart for imaging data (DCE-MRI & CVR).**


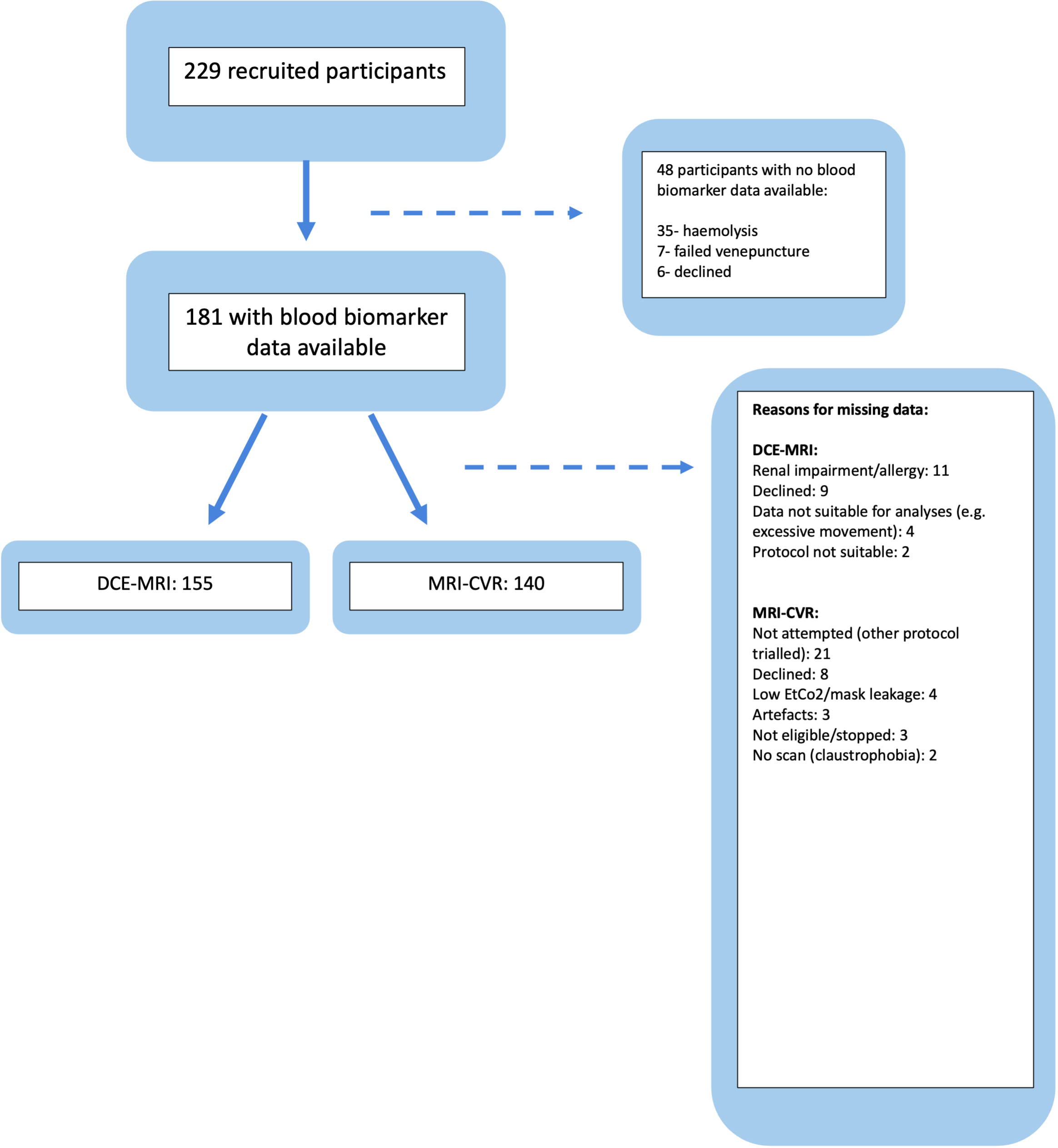


**Abbreviations:** **DCE-MRI:** dynamic contrast-enhanced magnetic resonance imaging; **CVR:** cerebrovascular reactivity; **EtCO_2_:** end-tidal carbon dioxide.

**Figure S2. Participant flow chart for cognitive data at baseline and 1 year (MoCA & TMT).**


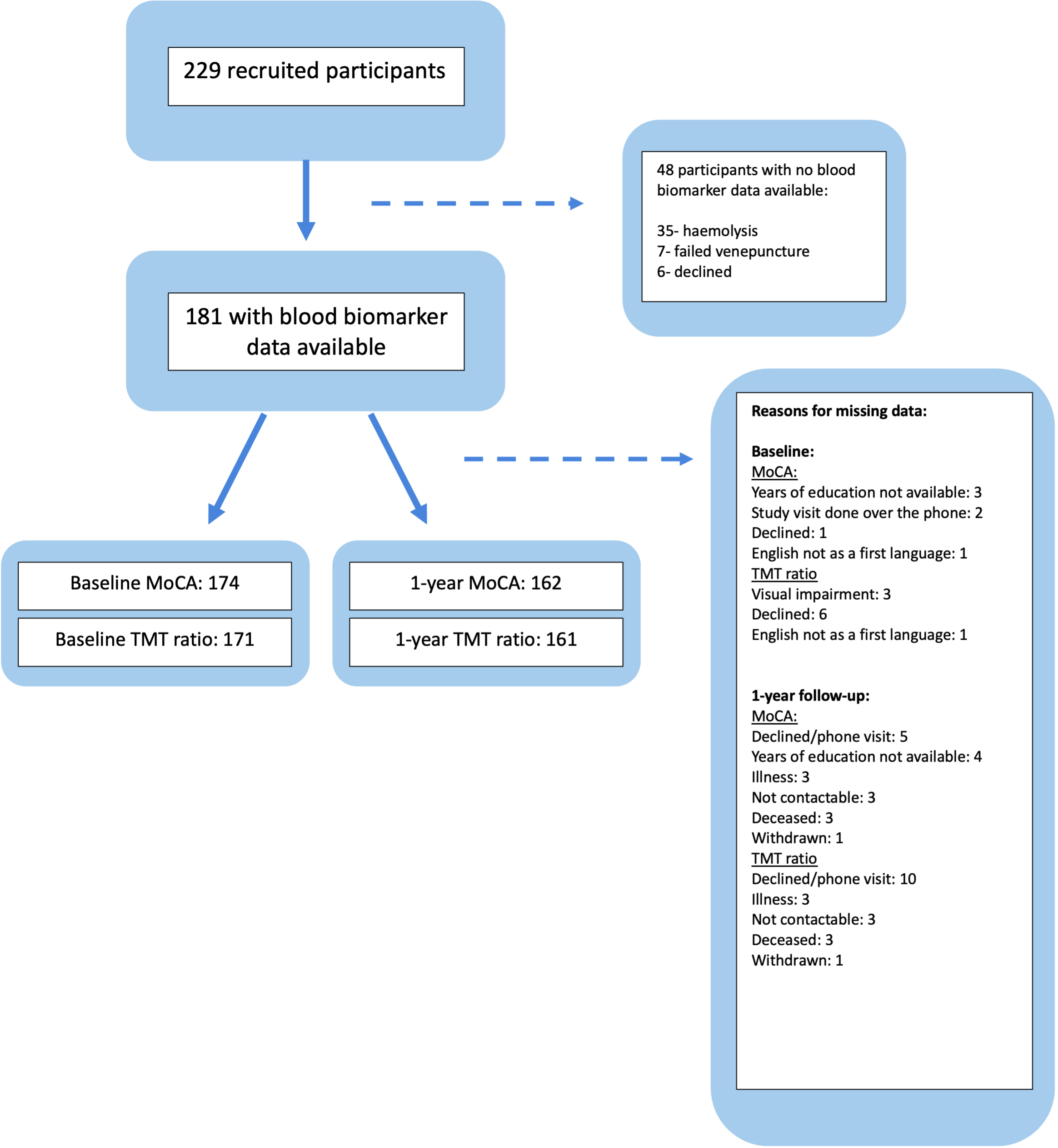


**Abbreviations:** **MoCA:** Montreal Cognitive Assessment; **TMT:** Trail Making Test.

**Figure S3. Blood biomarkers and WMH volume.** Regression results for the association between distinct standardised blood biomarkers and log transformed WMH volume as % ICV at baseline (linear regression) and one year follow-up (linear mixed effects models) after adjusting for age, sex, vascular risk factors, stroke subtype, and baseline NIHSS.

**
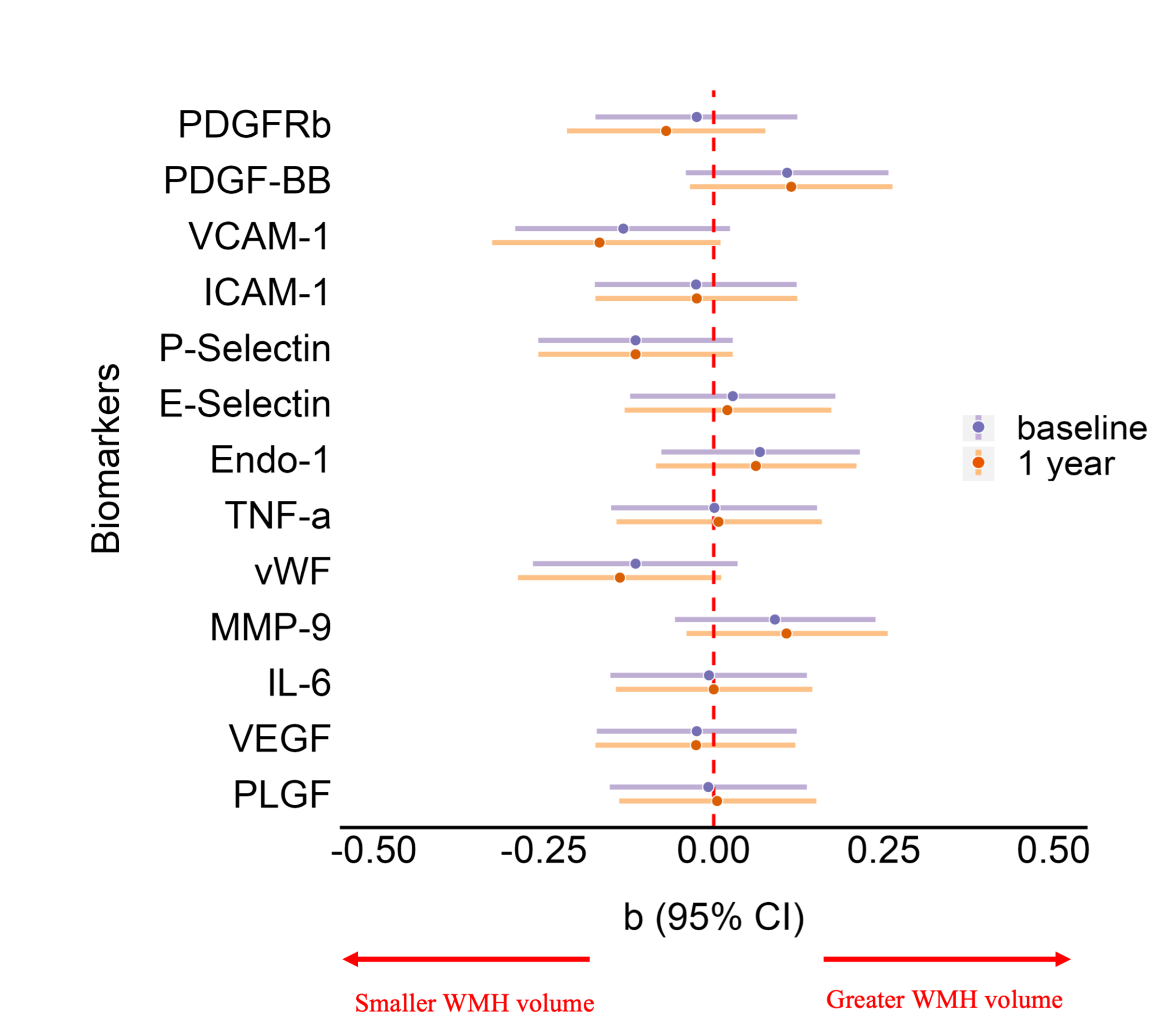
**

**Abbreviations:** **WMH:** white matter hyperintensity; **ICV:** intracranial volume; **NIHSS:** The National Institutes of Health Stroke Scale; **CI:** confidence interval; **PDGFRβ:** platelet derived growth factor receptor beta; **PDGF-BB:** platelet derived growth factor subunit B; **VCAM-1:** vascular cell adhesion molecule 1; **ICAM-1:** intercellular adhesion molecule 1; **E-selectin:** endothelial-selectin; **P-Selectin:** platelet-selectin; **Endo-1:** Endothelin-1; **TNF-α:** tumour necrosis factor alpha; **vWF:** von Willebrand Factor; **MMP-9:** matrix metalloproteinase 9; **IL-6:** Interleukin 6; **VEGF:** vascular endothelial growth factor; **PlGF:** placental growth factor.

**Figure S4. Blood biomarkers and Fazekas score.** Ordinal regression results for the association of distinct standardised blood biomarkers and Fazekas score at baseline after adjusting for age, sex, vascular risk factors, stroke subtype, and baseline NIHSS.


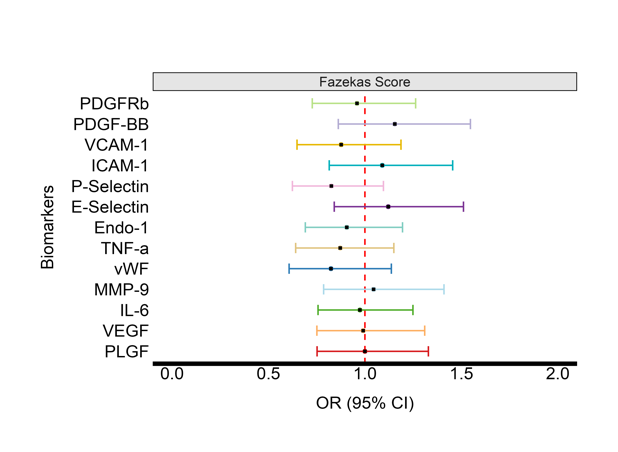


Lower Fazekas

Higher Fazekas

**Abbreviations:** **NIHSS:** The National Institutes of Health Stroke Scale; **CI:** confidence interval; **PDGFRβ:** platelet derived growth factor receptor beta; **PDGF-BB:** platelet derived growth factor subunit B; **VCAM-1:** vascular cell adhesion molecule 1; **ICAM-1:** intercellular adhesion molecule 1; **E-selectin:** endothelial-selectin; **P-Selectin:** platelet-selectin; **Endo-1:** Endothelin-1; **TNF-α:** tumour necrosis factor alpha; **vWF:** von Willebrand Factor; **MMP-9:** matrix metalloproteinase 9; **IL-6:** Interleukin 6; **VEGF:** vascular endothelial growth factor; **PLGF:** placental growth factor.

**Figure S5. Blood biomarkers and the presence of lacunes and/or microbleeds at baseline.** Logistic regression results for the association of distinct standardised blood biomarkers and the prevalence of lacunes and microbleeds at baseline after adjusting for age, sex, vascular risk factors, and stroke subtype.


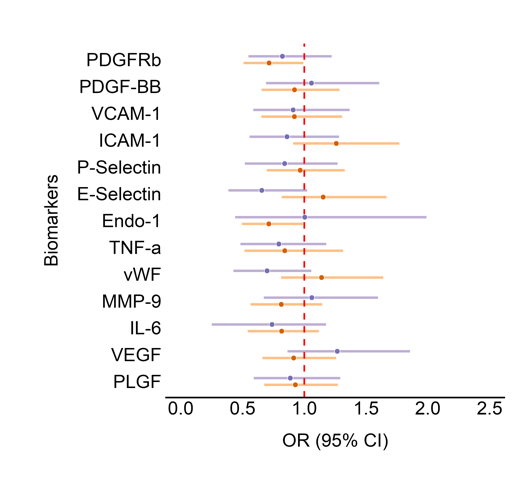


Lower

Higher


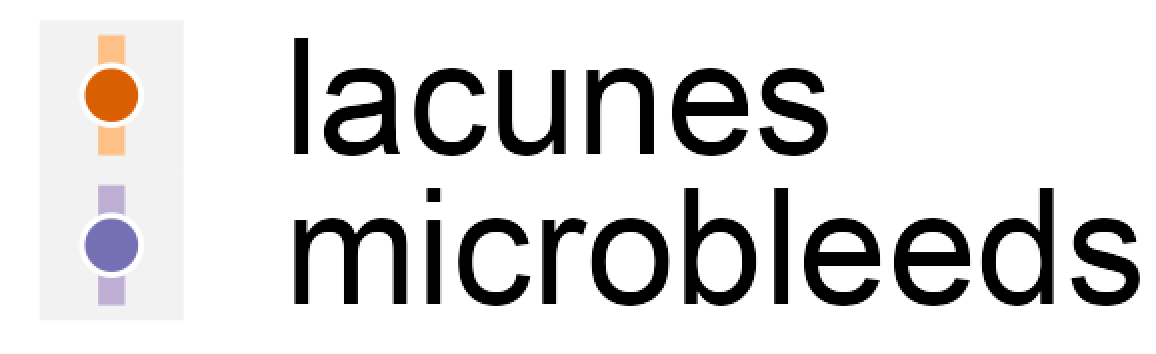

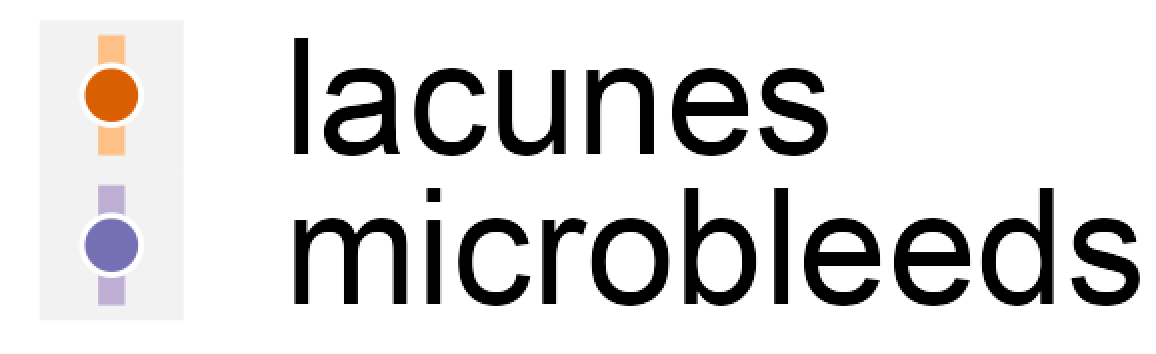


**Abbreviations:** **CI:** confidence interval; **PDGFRβ:** platelet derived growth factor receptor beta; **PDGF-BB:** platelet derived growth factor subunit B; **VCAM-1:** vascular cell adhesion molecule 1; **ICAM-1:** intercellular adhesion molecule 1; **E-selectin:** endothelial-selectin; **P-Selectin:** platelet-selectin; **Endo-1:** Endothelin-1; **TNF-α:** tumour necrosis factor alpha; **vWF:** von Willebrand Factor; **MMP-9:** matrix metalloproteinase 9; **IL-6:** Interleukin 6; **VEGF:** vascular endothelial growth factor; **PLGF:** placental growth factor.

**Figure S6. Blood biomarkers and summary SVD score.** Ordinal regression results for the association of distinct standardised blood biomarkers to baseline summary SVD scores after adjusting for age, sex, vascular risk factors, and stroke subtype.


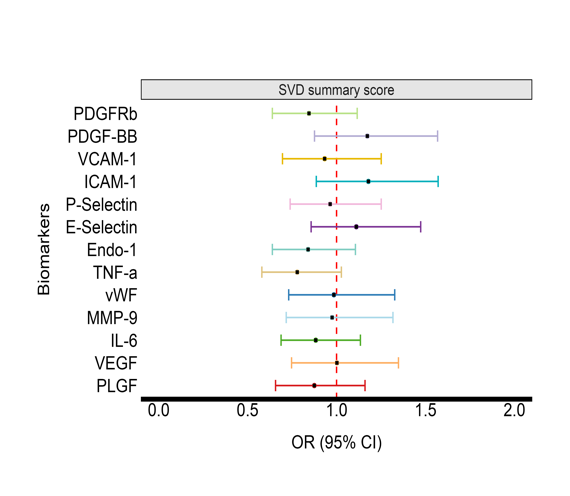


Lower SVD Score

Higher SVD Score

**Abbreviations:** **SVD:** small vessel disease; **CI:** confidence interval; **PDGFRβ:** platelet derived growth factor receptor beta; **PDGF-BB:** platelet derived growth factor subunit B; **VCAM-1:** vascular cell adhesion molecule 1; **ICAM-1:** intercellular adhesion molecule 1; **E-selectin:** endothelial-selectin; **P-Selectin:** platelet-selectin; **Endo-1:** Endothelin-1; **TNF-α:** tumour necrosis factor alpha; **vWF:** von Willebrand Factor; **MMP-9:** matrix metalloproteinase 9; **IL-6:** Interleukin 6; **VEGF:** vascular endothelial growth factor; **PLGF:** placental growth factor.

**Figure S7. Blood biomarkers and MCI.** Logistic regression results for the association of distinct standardised blood biomarkers and MCI prevalence at baseline and one year follow-up after adjusting for age, sex, vascular risk factors, premorbid intelligence, and baseline WMH volume as % ICV. One-year cross-sectional models were also adjusted for baseline MCI status.

**Abbreviations:** **MCI:** mild cognitive impairment; **WMH:** white matter hyperintensity; **ICV:** intracranial volume; **CI:** confidence interval; **PDGFRβ:** platelet derived growth factor receptor beta; **PDGF-BB:** platelet derived growth factor subunit B; **VCAM-1:** vascular cell adhesion molecule 1; **ICAM-1:** intercellular adhesion molecule 1; **E-selectin:** endothelial-selectin; **P-Selectin:** platelet-selectin; **Endo-1:** Endothelin-1; **TNF-α:** tumour necrosis factor alpha; **vWF:** von Willebrand Factor; **MMP-9:** matrix metalloproteinase 9; **IL-6:** Interleukin 6; **VEGF:** vascular endothelial growth factor; **PLGF:** placental growth factor.


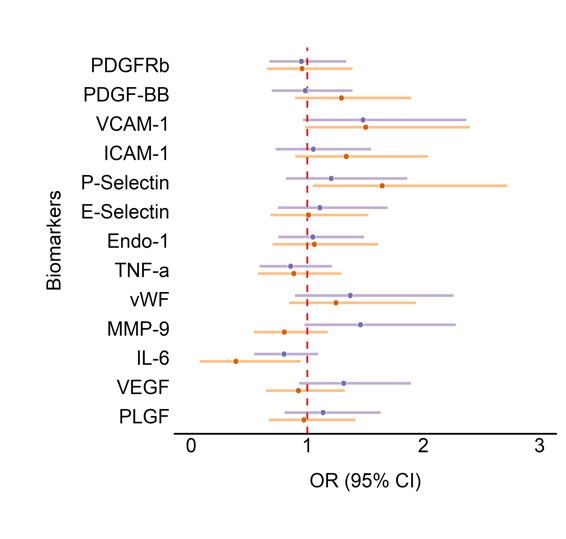

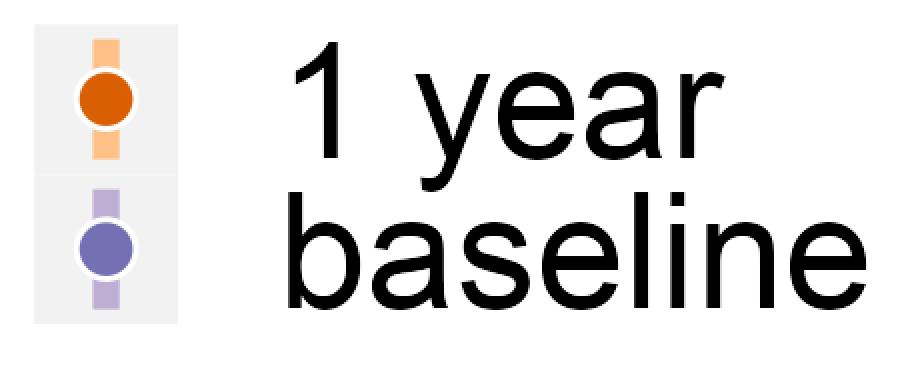

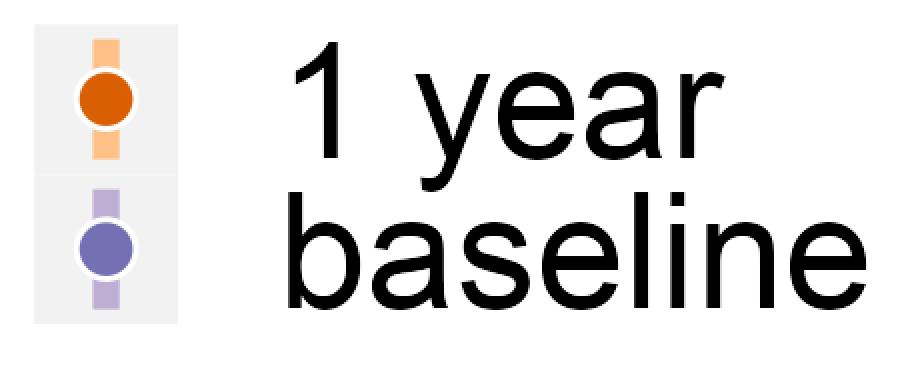


Lower

Higher

**Figure S8. Blood biomarkers and executive function (TMT ratio B/A).** Baseline cross-sectional linear regression and longitudinal linear mixed effects model results for the association of distinct standardised blood biomarkers and TMT ratio (B/A) after adjusting for age, sex, vascular risk factors, premorbid intelligence, and baseline WMH volume as % ICV.


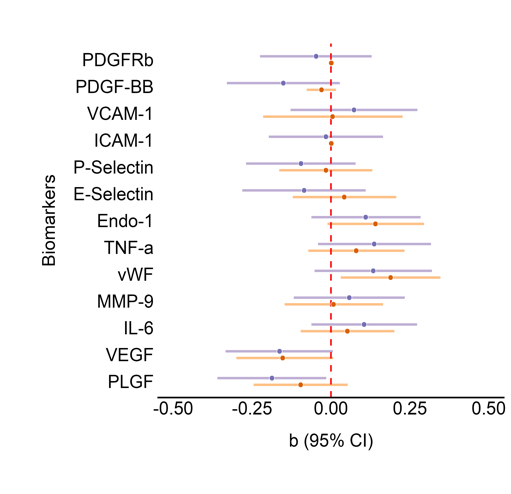

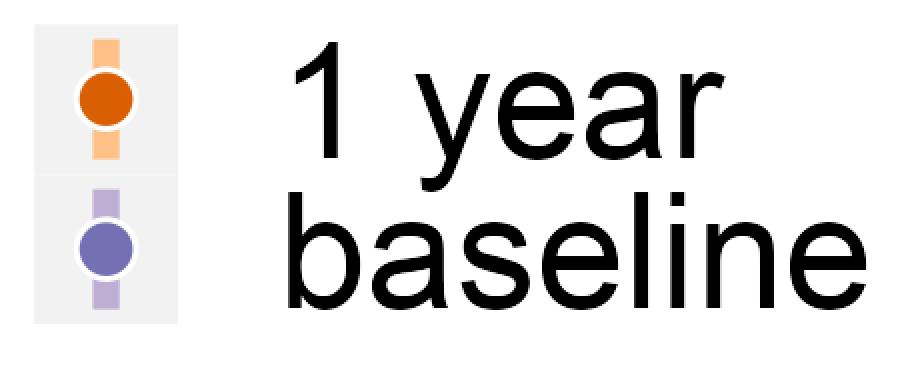

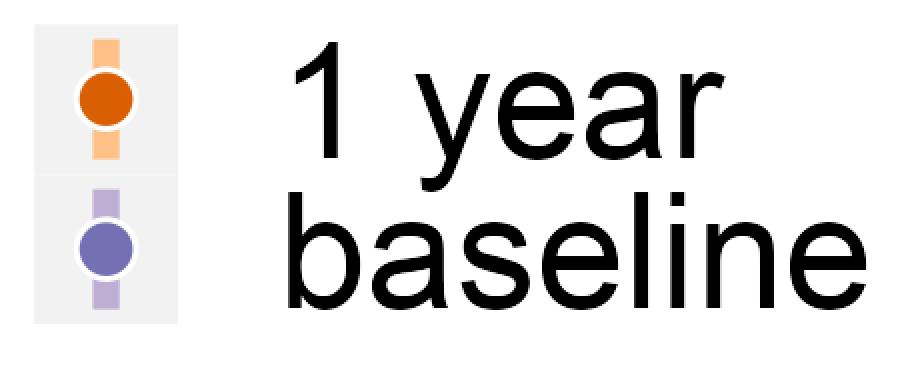


Better EF

Worse EF

**Abbreviations:** **TMT:** Trail Making Task; **WMH:** white matter hyperintensity; **ICV:** intracranial volume; **CI:** confidence interval; **PDGFRβ:** platelet derived growth factor receptor beta; **PDGF-BB:** platelet derived growth factor subunit B; **VCAM-1:** vascular cell adhesion molecule 1; **ICAM-1:** intercellular adhesion molecule 1; **E-selectin:** endothelial-selectin; **P-Selectin:** platelet-selectin; **Endo-1:** Endothelin-1; **TNF-α:** tumour necrosis factor alpha; **vWF:** von Willebrand Factor; **MMP-9:** matrix metalloproteinase 9; **IL-6:** Interleukin 6; **VEGF:** vascular endothelial growth factor; **PLGF:** placental growth factor; **EF:** executive function.

**Figure S9. Relationship between 13 biomarkers and 24 markers of disease grouped by effect type.** The first panel illustrates associations reported as beta coefficients from linear regression models, and the second shows associations reported as odds ratios from logistic regression models. Colour intensity reflects the strength of the relationship, with darker hues representing stronger effects. Blue shades indicate negative associations, whilst red shades represent positive associations. Significance is indicted by asterisks in the case where the confidence interval excludes 0 (for beta coefficients) or 1 (for odds ratios).


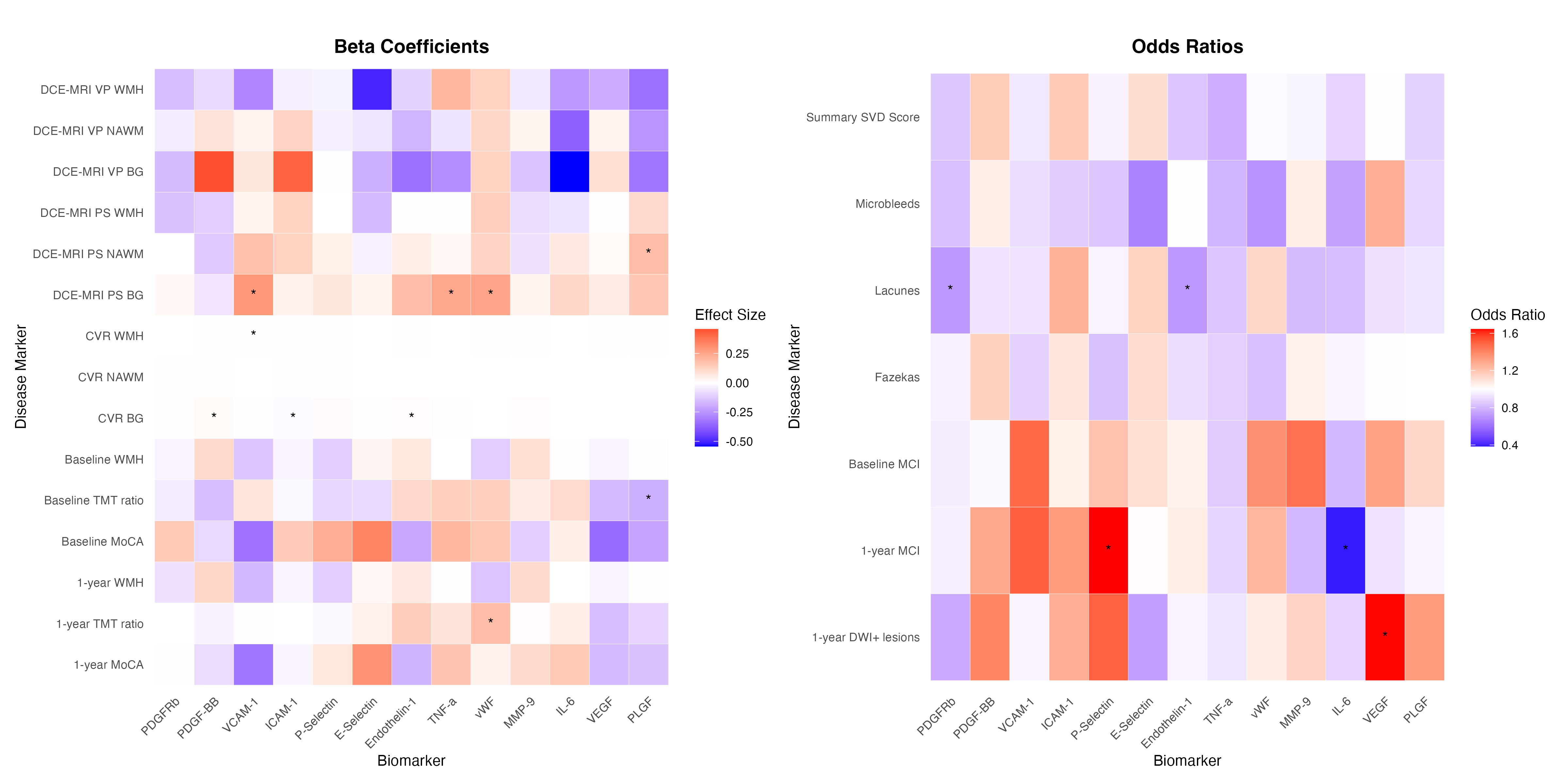


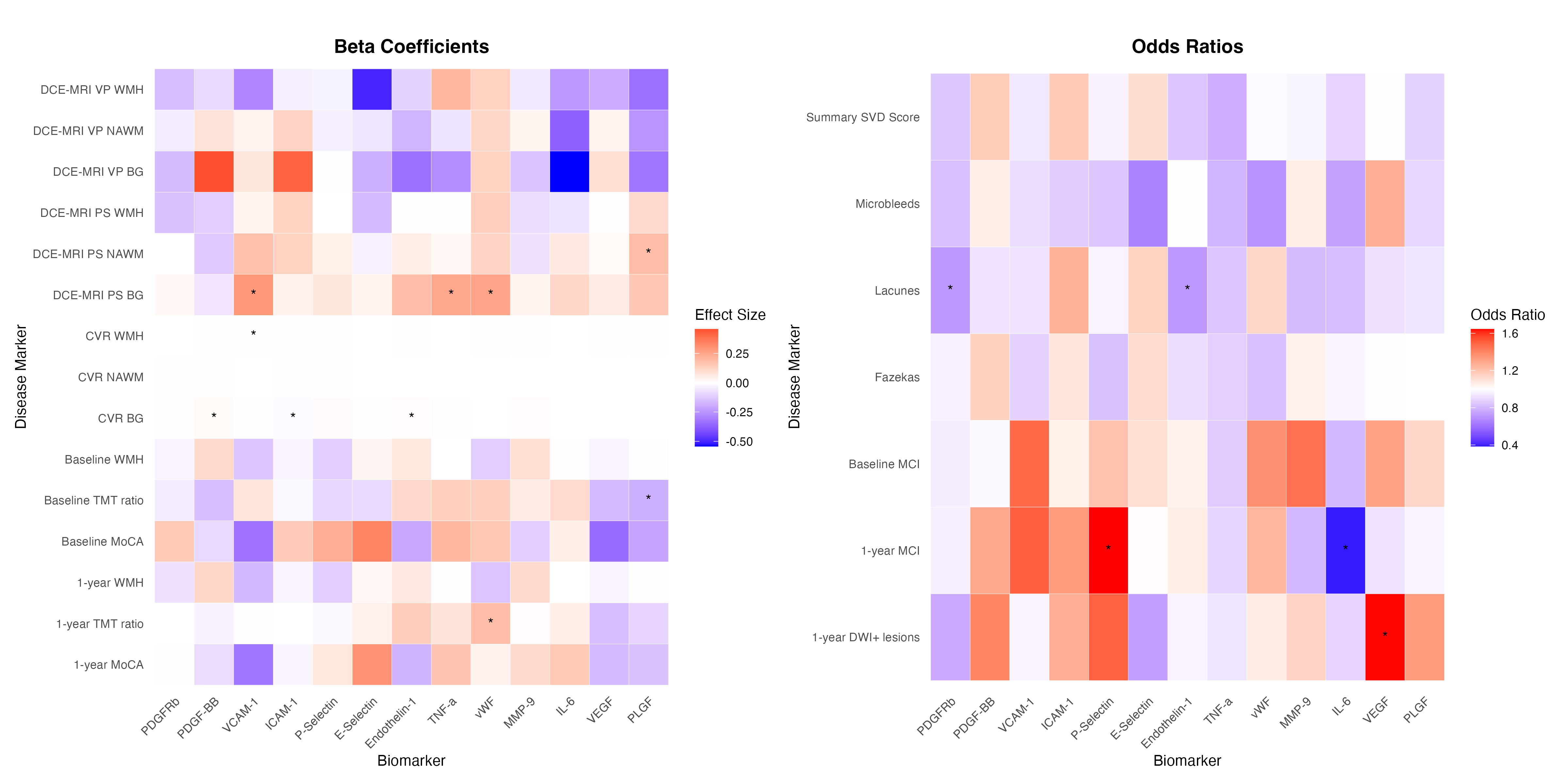


**Abbreviations:** **DCE-MRI:** dynamic contrast-enhanced magnetic resonance imaging; **V_p_:** blood plasma volume fraction; **WMH:** white matter hyperintensity; **NAWM:** normal appearing white matter; **BG:** basal ganglia; **PS:** Permeability-Surface area product; **CVR:** cerebrovascular reactivity; **TMT:** Trail Making Task; **MoCA:** Montreal cognitive Assessment; **PDGFRβ:** platelet derived growth factor receptor beta; **PDGF-BB:** platelet derived growth factor subunit B; **VCAM-1:** vascular cell adhesion molecule 1; **ICAM-1:** intercellular adhesion molecule 1; **E-selectin:** endothelial-selectin; **P-Selectin:** platelet-selectin; **Endo-1:** Endothelin-1; **TNF-α:** tumour necrosis factor alpha; **vWF:** von Willebrand Factor; **MMP-9:** matrix metalloproteinase 9; **IL-6:** Interleukin 6; **VEGF:** vascular endothelial growth factor; **PLGF:** placental growth factor; **SVD:** small vessel disease; **MCI:** mild cognitive impairment; **DWI:** diffusion-weighted imaging.

**Supplemental Appendix A. Blood biomarker ELISA protocol, kits, and dilutions**

**PDGFRβ**

Kit: Abcam ab252357. Lot number: 2101037333

Initial testing: 4 samples (3xserum, 1xplasma) were tested on the kit at neat, x5 and x25 dilutions. From this it was decided that serum samples would all be tested at x10 dilution, and plasma samples at x4 dilution.

Samples were tested as described; some samples required a re-test. Serum samples that were re-tested were performed at x10 and x100 dilutions, plasma samples at x20 and x50 dilutions. There was no provided control to perform an inter-assay % CV. The intra-assay % CV was calculated as 3.12%.

**PDGF-BB**

Kit: Abcam ab100624. Lot number: 1010637-1

Initial testing: 4 samples (3xserum, 1xplasma) were tested on the kit at neat, x5, x50 and x500 dilutions. From this it was decided that serum samples would all be tested at x100 dilution, and plasma samples at x20 dilution.

Samples were tested as described, and no re-tests were required. There was no provided control to perform an inter-assay % CV. The intra-assay % CV was calculated as 1.98%.

**VCAM-1**

Kit: Abcam ab223591. Lot number: 2101036376

Initial testing: 4 samples (3xserum, 1xplasma) were tested on the kit at neat, x5, x50 and x500 dilutions. From this it was decided that all samples would be tested at x500 dilution.

Samples were tested as described; some samples required a re-test. Samples that were re-tested were performed at x500, x1000, x2000 and x4000 dilutions. There was no provided control to perform an inter-assay % CV. The intra-assay % CV was calculated as 5.90%.

**ICAM-1**

Kit: R&D DY720. Lot number: P357734

Initial testing: 4 samples (3xserum, 1xplasma) were tested on the kit at neat, x5, x50 and x500 dilutions. From this it was decided that all samples would be tested at x500 dilution.

Samples were tested as described, and no re-tests were required. There was no provided control to perform an inter-assay % CV. The intra-assay % CV was calculated as 3.88%.

**P-Selectin**

Kit: R&D DPSE00. Lot number: P357395

Initial testing: 4 samples (3xserum, 1xplasma) were tested on the kit at x20, x60 and x120 dilutions. From this it was decided that all samples would be tested at x20 dilution.

Samples were tested as described, and no re-tests were required. A control was provided with the kit, the inter-assay % CV was calculated as 15.63%. The intra-assay % CV was calculated as 3.84%.

**E-Selectin**

Kit: R&D DSLE00. Lot number: P367030

Controls: QC236. Lot number: P314314

Initial testing: 3 samples (3xserum; kit not suitable for plasma samples) were tested on the kit at neat, x10 and x100 dilutions. From this it was decided that all samples would be tested at x10 dilution.

Samples were tested as described; some samples required a re-test. Samples that were re-tested were performed at x10 and x100 dilutions. The inter-assay % CV was calculated as 8.08%. The intra-assay % CV was calculated as 3.75%.

**Endothelin-1**

Kit: R&D DET100. Lot number: P357232

Controls: QC82. Lot numbers: 1663979, 1663980, 1663982 (1 per control level)

Initial testing: 4 samples (3xserum, 1xplasma) were tested on the kit at neat, x5, x50 and x500 dilutions. From this it was decided that all samples would be tested neat.

Samples were tested as described, and no re-tests were required. The inter-assay % CV was calculated as 11.21%. The intra-assay % CV was calculated as 16.73%.

**TNF-α**

Kit: R&D HSTA00E. Lot number: P348076

Controls: QC232. Lot number: P335817

Initial testing: 4 samples (3xserum, 1xplasma) were tested on the kit at neat, x5, x50 and x500 dilutions. From this it was decided that all samples would be tested neat.

Samples were tested as described, and no re-tests were required. The inter-assay % CV was calculated as 13.77%. The intra-assay % CV was calculated as 7.06%.

**vWF**

Kit: Abcam ab108918. Lot number: 1011742-2

Initial testing: 4 samples (3xserum, 1xplasma) were tested on the kit at neat, x10, x100 and x400 dilutions. From this it was decided that all samples would be tested at x100 dilution.

Samples were tested as described; some samples required a re-test. Samples that were re-tested were performed at x100, x200 and x600 dilutions. There was no provided control to perform an inter-assay % CV. The intra-assay % CV was calculated as 6.79%.

**MMP-9**

Kit: R&D DMP900. Lot number: P363877

Controls: QC130. Lot number: P319142

Initial testing: 3 samples (3xserum; kit not suitable for plasma samples) were tested on the kit at neat, x10 and x400 dilutions. From this it was decided that all samples would be tested at x100 dilution.

Samples were tested as described; some samples required a re-test. Samples that were re-tested were performed at x100, x200 and x600 dilutions. The inter-assay % CV was calculated as 3.68%. The intra-assay % CV was calculated as 5.48%.

**IL-6**

Kit: R&D HS600C. Lot number: P355671

Controls: QC246. Lot number: P357243

Initial testing: 4 samples (3xserum, 1xplasma) were tested on the kit at neat, x5, x50 and x500 dilutions. From this it was decided that all samples would be tested neat.

Samples were tested as described; some samples required a re-test. Samples that were re-tested were performed at x5 and x50 dilutions. The inter-assay % CV was calculated as 15.71%. The intra-assay % CV was calculated as 10.10%.

**VEGF**

Kit: R&D DVE00. Lot number: P350540

Controls: QC01-1. Lot number: P343594

Initial testing: 4 samples (3xserum, 1xplasma) were tested on the kit at neat, x5, x50 and x500 dilutions. From this it was decided that all samples would be tested neat.

Samples were tested as described, and no re-tests were required. The inter-assay % CV was calculated as 39.68%. The intra-assay % CV was calculated as 13.26%.

**PlGF**

Kit: R&D HSPG00. Lot number: P382640

Controls: QC144

Initial testing: 4 samples (3xserum, 1xplasma) were tested on the kit at neat, x10 and 100 dilutions. From this it was decided that all samples would be tested neat.

Samples were tested as described, and no re-tests were required. The inter-assay % CV was calculated as 11.35%. The intra-assay % CV was calculated as 12.31%.
