# Supplementary material for: Blood biomarkers of vascular dysfunction in small vessel disease progression: Insights from a longitudinal neuroimaging study
Source: Alzheimers Dement. 2025 Apr 25;21(4):e70152. doi: 10.1002/alz.70152 (PMC12022501; doi:10.1002/alz.70152)
Supplement: Supplementary file 3 — Supporting Information [file ALZ-21-e70152-s001.docx]

# Supplemental material

Supplementary material is available online.

# Appendix

The Mild Stroke Study 3 Group (MSS3)

| **Member** | **Affiliations** |
| --- | --- |
| Charlotte Jardine | Edinburgh Imaging Facility (Royal Infirmary of Edinburgh), University of Edinburgh, Edinburgh EH16 4SA, UK |
| Rachel Locherty | Centre for Clinical Brain Sciences, University of Edinburgh, Edinburgh EH16 4SB, UK;  UK Dementia Research Institute, University of Edinburgh, Edinburgh EH16 4SB, UK |
| Iona Hamilton | Edinburgh Imaging Facility (Royal Infirmary of Edinburgh), University of Edinburgh, Edinburgh EH16 4SA, UK |
| Alasdair Morgan | Centre for Clinical Brain Sciences, University of Edinburgh, Edinburgh EH16 4SB, UK;  UK Dementia Research Institute, University of Edinburgh, Edinburgh EH16 4SB, UK |
| Cameron Manning | Centre for Clinical Brain Sciences, University of Edinburgh, Edinburgh EH16 4SB, UK;  UK Dementia Research Institute, University of Edinburgh, Edinburgh EH16 4SB, UK |
| Agniete Kampaite | Centre for Clinical Brain Sciences, University of Edinburgh, Edinburgh EH16 4SB, UK |
| Eleni Sakka | Centre for Clinical Brain Sciences, University of Edinburgh, Edinburgh EH16 4SB, UK |
| Olivia K.L.  Hamilton | Centre for Clinical Brain Sciences, University of Edinburgh, Edinburgh EH16 4SB, UK;  UK Dementia Research Institute, University of Edinburgh, Edinburgh EH16 4SB, UK;  MRC/CSO Social and Public Health Sciences Unit, School of Health and Wellbeing, University of Glasgow, Clarice Pears Building, 90 Byres Road, Glasgow, G12 8TB |
| Will Hewins | Centre for Clinical Brain Sciences, University of Edinburgh, Edinburgh EH16 4SB, UK;  UK Dementia Research Institute, University of Edinburgh, Edinburgh EH16 4SB, UK |
| Xiaodi Liu | Centre for Clinical Brain Sciences, University of Edinburgh, Edinburgh EH16 4SB, UK;  Division of Neurology, Department of Medicine, LKS Faculty of Medicine, The University of Hong Kong, Hong Kong SAR, China |
| Kirsten Wilson | Centre for Reproductive Health, Institute for Regeneration and Repair, University of Edinburgh, Edinburgh, EH16, 4UU, UK. |
| Donna McIntyre | Edinburgh Imaging Facility (Royal Infirmary of Edinburgh), University of Edinburgh, Edinburgh EH16 4SA, UK |
| Gayle Barclay | Edinburgh Imaging Facility (Royal Infirmary of Edinburgh), University of Edinburgh, Edinburgh EH16 4SA, UK |
| Lucy Clara Kesseler | Edinburgh Imaging Facility (Royal Infirmary of Edinburgh), University of Edinburgh, Edinburgh EH16 4SA, UK |
| Madeleine Murphy | Edinburgh Imaging Facility (Royal Infirmary of Edinburgh), University of Edinburgh, Edinburgh EH16 4SA, UK |
| Sean Denham | Edinburgh Imaging Facility (Royal Infirmary of Edinburgh), University of Edinburgh, Edinburgh EH16 4SA, UK |
